# Adhesion of Bartonella henselae to Fibronectin Is Mediated via Repetitive Motifs Present in the Stalk of *Bartonella* Adhesin A

**DOI:** 10.1128/spectrum.02117-22

**Published:** 2022-09-27

**Authors:** Arno Thibau, Diana J. Vaca, Marlene Bagowski, Katharina Hipp, Daniela Bender, Wibke Ballhorn, Dirk Linke, Volkhard A. J. Kempf

**Affiliations:** a Institute for Medical Microbiology and Infection Control, University Hospital, Goethe University, Frankfurt, Germany; b Electron Microscopy Facility, Max Planck Institute for Developmental Biology, Tübingen, Germany; c Federal Institute for Vaccines and Biomedicines, Department of Virology, Paul-Ehrlich-Institut, Langen, Germany; d Section for Genetics and Evolutionary Biology, Department of Biosciences, University of Oslo, Oslo, Norway; University of Wollongong

**Keywords:** *Bartonella* adhesin A, trimeric autotransporter adhesin, fibronectin, pathogenicity, bacterium-host interaction

## Abstract

Adhesion to host cells is the first and most crucial step in infections with pathogenic Gram-negative bacteria and is often mediated by trimeric autotransporter adhesins (TAAs). Bartonella henselae targets the extracellular matrix glycoprotein fibronectin (Fn) via the *Bartonella* adhesin A (BadA) attaching the bacteria to the host cell. The TAA BadA is characterized by a highly repetitive passenger domain consisting of 30 neck/stalk domains with various degrees of similarity. To elucidate the motif sequences mediating Fn binding, we generated 10 modified BadA constructs and verified their expression via Western blotting, confocal laser scanning, and electron microscopy. We analyzed their ability to bind human plasma Fn using quantitative whole-cell enzyme-linked immunosorbent assays (ELISAs) and fluorescence microscopy. Polyclonal antibodies targeting a 15-mer amino acid motif sequence proved to reduce Fn binding. We suggest that BadA adheres to Fn in a cumulative effort with quick saturation primarily via unpaired β-strands appearing in motifs repeatedly present throughout the neck/stalk region. In addition, we demonstrated that the length of truncated BadA constructs correlates with the immunoreactivity of human patient sera. The identification of BadA-Fn binding regions will support the development of new “antiadhesive” compounds inhibiting the initial adherence of B. henselae and other TAA-expressing pathogens to host cells.

**IMPORTANCE** Trimeric autotransporter adhesins (TAAs) are important virulence factors and are widely present in various pathogenic Gram-negative bacteria. TAA-expressing bacteria cause a wide spectrum of human diseases, such as cat scratch disease (Bartonella henselae), enterocolitis (Yersinia enterocolitica), meningitis (Neisseria meningitis), and bloodstream infections (multidrug-resistant Acinetobacter baumannii). TAA-targeted antiadhesive strategies (against, e.g., *Bartonella* adhesin A [BadA], *Yersinia* adhesin A [YadA], *Neisseria* adhesin A [NadA], and Acinetobacter trimeric autotransporter [Ata]) might represent a universal strategy to counteract such bacterial infections. BadA is one of the best characterized TAAs, and because of its high number of (sub)domains, it serves as an attractive adhesin to study the domain-function relationship of TAAs in the infection process. The identification of common binding motifs between TAAs (here, BadA) and their major binding partner (here, fibronectin) provides a basis toward the design of novel “antiadhesive” compounds preventing the initial adherence of Gram-negative bacteria in infections.

## INTRODUCTION

Adhesion is the primary and most crucial step in infections with pathogens. In Gram-negative bacteria, trimeric autotransporter adhesins (TAAs; type Vc secretion system) represent an important class of adhesins. They display a common architecture consisting of a long N-terminal passenger domain and a highly conserved C-terminal anchor domain (1, 2). TAAs are present widely in various human-pathogenic Gram-negative bacteria, of which *Yersinia* adhesin A (YadA) of Yersinia enterocolitica is considered the prototypical TAA. Other well-known examples are Acinetobacter trimeric autotransporter (Ata) of Acinetobacter baumannii, *Neisseria* adhesin A (NadA) of Neisseria meningitidis, and Salmonella adhesin A (SadA) of Salmonella enterica (3–6). One of the best characterized TAAs is *Bartonella* adhesin A (BadA) of B. henselae that is essential for bacterial binding to extracellular matrix (ECM) proteins (e.g., collagen, laminin, and fibronectin [Fn]) and endothelial cells and that facilitates angiogenic reprograming of infected host cells (7, 8). It was described recently that the interaction of B. henselae with Fn represents the molecular basis for adhesion to host cells by attaching the bacteria to the host cell surface via an “Fn-bridge” (9).

B. henselae is a fastidious, facultative intracellular, and zoonotic pathogen. Cats are the main reservoir host of B. henselae, and infections result primarily in a long-lasting asymptomatic bacteremia (10, 11). The cat flea (Ctenocephalides felis) serves as the major vector for transmission among cats (12). Incidental infection of immunocompetent humans usually results in cat scratch disease, a self-limiting illness often manifesting as localized lymphadenopathy. However, immunocompromised patients might suffer from life-threating endocarditis (13, 14) or vasculoproliferative disorders, such as bacillary angiomatosis (15, 16).

The modularly structured passenger domain of BadA includes a head domain and an exceptionally long neck/stalk region showing high variability among different B. henselae strains (17, 18). Using state-of-the-art long-read sequencing technologies, we demonstrated recently that B. henselae Marseille contains a *badA* gene of 11,922 bp with a neck/stalk region that contains 30 repetitive domains (17). Neck/stalk domains share specific sequence motifs with characteristic conformations, annotated by the “domain dictionary” approach of the daTAA (domain annotation of TAAs) server (19, 20). The head domain of BadA is responsible for the majority of its biological functions (21, 22), whereas adhesion to Fn is mediated via the neck/stalk region (23). Fn is a heterodimeric glycoprotein that is present abundantly on the cell surface of endothelial cells (cellular Fn) or in blood and other fluids (plasma Fn) (24, 25), making it an excellent initial binding partner in infections of blood vessels, heart valves, or in the case of a cat scratch in the human skin.

Interaction sites between Fn and BadA have been mapped out previously via cross-linking mass spectrometry (XL-MS) (9). However, the exact motifs within the various BadA domains responsible for Fn binding remain unknown. In this study, we identified specific repeated motif sequences involved in adherence to Fn using bacterial binding assays and truncated and modified BadA constructs expressed on the surface of a BadA-deficient B. henselae transposon mutant strain (ΔBadA-T). These findings might contribute to the development of new “antiadhesive” compounds inhibiting BadA-mediated binding of B. henselae to endothelial cells in the initial course of infection and provide a basis for further research in the design of general “anti-infective” therapeutic strategies against other TAA-expressing pathogenic bacteria.

## RESULTS

### The BadA neck/stalk region consists of repetitive motifs with various degrees of similarity.

**FIG 1:**
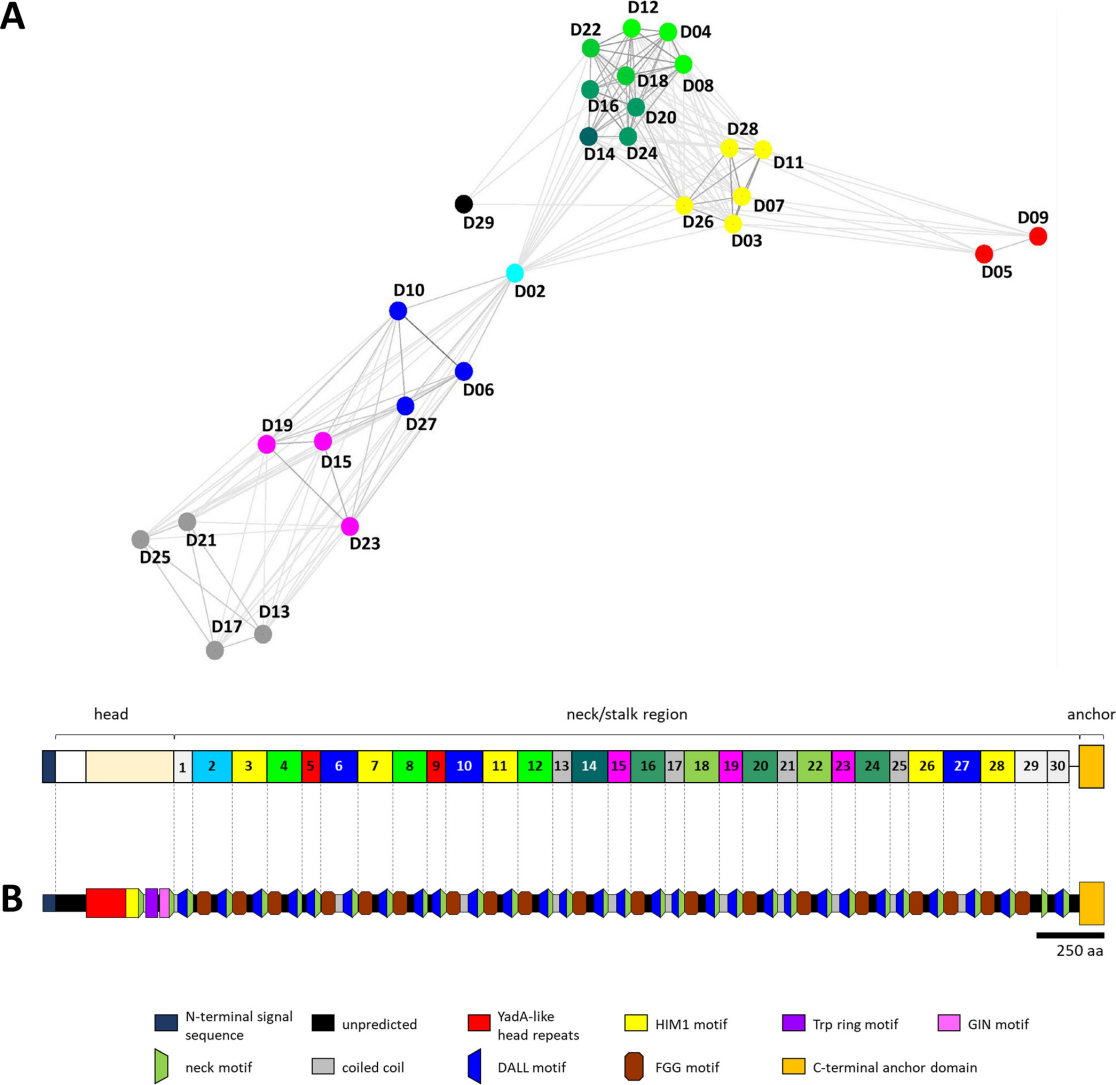
*In silico* prediction and schematic domain organization of the complete BadA fiber of B. henselae Marseille. (A) BadA is modularly structured and is organized in 30 neck/stalk domains defined by their respective neck motif sequence. Displayed colors represent domains with a high amino acid sequence similarity as is demonstrated by the pairwise domain similarity plot created in a three-dimensional space using CLANS (48). Domains 1 and 30 are not included because of their highly divergent pairwise domain similarity. (B) Motif sequences are annotated using the domain dictionary approach of the daTAA server (19). BadA contains an N-terminal head domain, including YadA-like head repeats, a HIM1 motif, a TRP ring motif, and a GIN motif. The long and repetitive neck/stalk region is organized in a recurring pattern, including an FGG motif, a coil-coiled motif, and a DALL-neck tandem connector. The C-terminal anchor domain is highly conserved among the family of TAAs, consists of a 12-stranded β-barrel, and is embedded in the outer membrane (20). (A and B) BadA images are drawn to scale according to amino acid sequence length. Scale bar: 250 aa.

**FIG 2:**
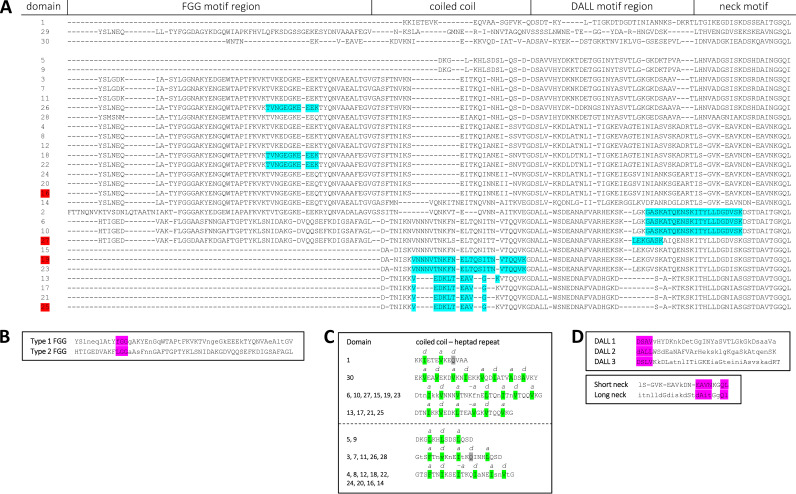
Protein sequence alignment of the BadA neck/stalk domains of B. henselae Marseille via daTAA server-defined sequence motifs. (A) The repetitive and modular architecture of BadA is demonstrated through the alignment of sequence motifs using the daTAA server (19). Previously identified cross-linking mass spectrometry Fn-BadA interaction sites (9) are highlighted in light blue. Red-highlighted domains were used to generate the modified BadA mutants (i.e., D16S28, D19S28, D25S28, and D27S29). (B) Two comparable FGG motif subtypes are observed within 19 of 30 neck/stalk domains and are characterized by a signature “(F/L)GG” sequence (purple). (C) Motif sequences above the dotted line are predicted *in silico* to encode a coiled-coil and comprise a heptad repeat. Hydrophobic residues are highlighted green (*a* and *d*) and are consistently separated by two and three polar residues. Hydrophilic residues are highlighted in gray. Sequence motifs below the dotted line are predicted *in silico* to encode α-helix structures, and yet a similar heptad repeat can be observed. (D) The DALL-neck tandem connector is present at the end of each neck/stalk domain and includes an α-to-β-to-α unit. Three variants of the DALL motif exist and are characterized by signature sequences “DSAV”, “DALL”, and “DSLV” (purple). Neck motif sequences appear as either long (22 aa) or short (19 aa) variants and show a common signature motif (purple). (B to D) Depicted residues in consensus sequences are uppercase if present in all observed motifs or in lowercase if they represent the relatively most frequent residues.

The modularly structured neck/stalk region of BadA consists of highly intertwined domains that are sequentially strung up (like beads on a string). Based on amino acid (aa) pairwise similarity (analyzed using cluster analysis of sequences [CLANS]), the 30 neck/stalk domains can be clustered into groups within a three-dimensional plot (Fig. 1A). Each neck/stalk domain is defined by a respective neck motif sequence and can be further organized into shorter sequence motifs using the domain dictionary approach of the daTAA server (Fig. 1B) (19). Interdomain sequence similarities and frequent repetitions of certain motif sequences are observed throughout BadA. A typical domain architecture within the neck/stalk region consists of an FGG motif sequence for larger domains (>87 amino acids), followed by a coiled-coil motif sequence, a DALL motif sequence, and a final neck motif sequence (Fig. 1B and 2A).

FGG motifs come as different subtypes but are described as an insertion of a 3-stranded β-meander into a coiled-coil region causing a 120° twist of the subunit chains around the trimer axis and generally support the long BadA trimer against vertical shear stress (3). The multiple glycine residues in the FGG motif sequence facilitate these tight turns in the trimeric structure and the formation of short loops (26). BadA contains two types of “(F/L)GG” motif sequences (Fig. 2B), and both types show similarity to the motif originally observed in SadA of S. enterica (20). Only domains 6, 10, and 27 include a type 2 FGG motif. In total, 19 FGG motifs are present, and only the smaller neck/stalk domains (≤87 aa) lack such a sequence.

Both FGG and neck motifs consistently precede a coiled-coil region, characterized by a superhelical α-helix structure (Fig. 2C). Coiled-coils generally have a “knobs-into-holes” packing where hydrophobic residues from one strand are situated in the trimeric core and fit into a space enveloped by the other two strands (27). Most motifs (i.e., in domains 1, 6, 10, 13, 15, 17, 19, 21, 23, 25, 27, and 30) consist of a heptad repeat where the seven amino acid positions are labeled “*abcdefg*,” with *a* and *d* commonly being the hydrophobic residues buried in the knob and separated by primarily polar residues, mainly present on the trimer’s exterior (20). A resolved crystal structure of the BadA head, including part of the neck/stalk region, shows clearly that a coiled-coil segment is present in domain 1 (28). Exceptions or unusual compositions, such as polar core residues or interrupted heptad repeats, are observed rarely in the BadA neck/stalk domains (Fig. 2C).

Transitions from an α-helix to β-stranded structures and back to an α-helix are facilitated via DALL and neck motif sequences, respectively (20). The DALL-neck tandem connector is present at the end of each BadA neck/stalk domain (Fig. 1B and 2A), consists of β-sheets forming a hairpin, and is considered to be a conformationally flexible region (29). BadA includes three variants of DALL motif sequences, with each present in approximately equal numbers with signature sequences “DSAV,” “DALL,” and “DSLV” (Fig. 2D). Neck motif sequences appear as either long (22 aa) or short (19 aa) variants. Long neck motifs are preceded consistently by a signature “DSAV” or “DALL” motif sequence, while the signature “DSLV” motif is always followed by a short neck motif.

### Modified and truncated BadA mutants are expressed on the surface of B. henselae ΔBadA-T.

**FIG 3:**
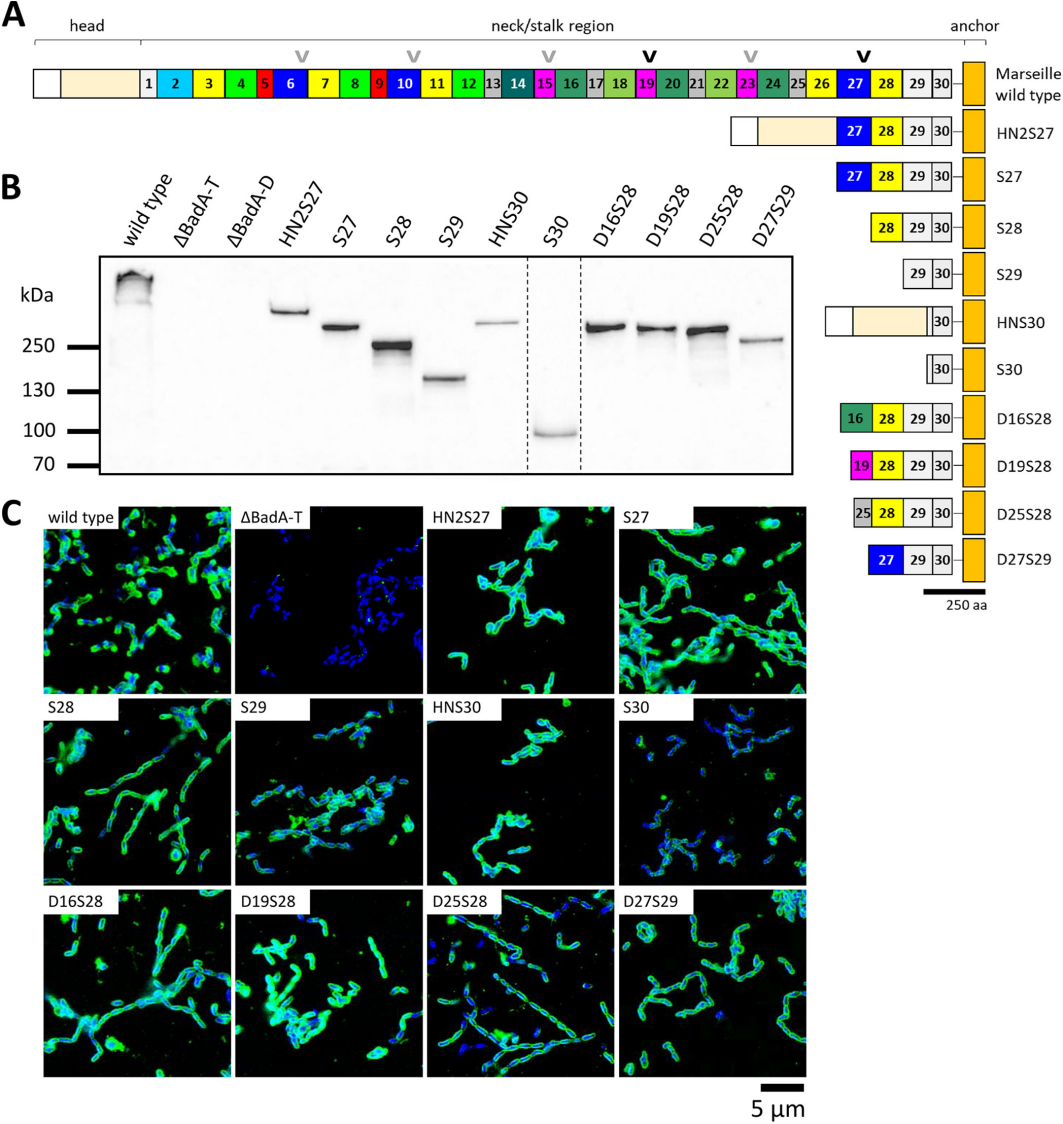
Overview of the generated BadA mutants and analysis of their protein expression via Western blotting and immunofluorescence confocal laser scanning microscopy. (A) Truncated and modified BadA proteins, shown underneath the wild-type BadA, are constructed by removing a certain number of neck/stalk domains. BadA S27 and S30 are the headless variants of HN2S27 and HNS30, respectively. BadA mutants D16S28, D19S28, D25S28, and D27S29 are all generated by combining a specific neck/stalk domain with BadA S28 or S29 as a basis. Arrowheads indicate a common motif sequence (LEKGASKATQENSKITYLLDGDVSK) present within the DALL-neck tandem connector of domains 19 and 27 (black) and highly similar domains (gray) that are presumed to mediate Fn binding. Images are drawn to scale according to aa sequence length. Scale bar: 250 aa. (B) The variety in molecular weight (MW) of all BadA mutant proteins is demonstrated via Western blotting using anti-BadA IgG antibodies (17). B. henselae strains HN2S27 and S30 display the largest (predicted as 343 kDa) and smallest (92 kDa) truncated BadA, respectively. Strain B. henselae Marseille wild type shows the highest MW band (predicted as 1,251 kDa), while negative-control strains ΔBadA-T and ΔBadA-D lack the ability to express BadA. Bacteria were analyzed on two separate nitrocellulose membranes in which the order of columns has been rearranged *in silico* (dotted line). (C) BadA surface localization was analyzed via CLSM using anti-BadA IgG antibodies and is indicated by a green halo around the DAPI-stained intracellular DNA. Negative-control strain B. henselae Marseille ΔBadA-T does not exhibit a green halo. Scale bar: 5 μm.

To investigate the role of individual neck/stalk BadA domains in their ability to bind the extracellular matrix protein Fn, we exploited the modular TAA architecture and transformed B. henselae ΔBadA-T with various BadA mutants (Fig. 3A). Mutant strains and BadA constructs will be mentioned by their plasmid name from here on (e.g., B. henselae ΔBadA-T/pS27 becomes strain S27). For clarification, S refers to stalk, H refers to head, N refers to neck, D refers to domain, and subsequent numbers denote the first N-terminal domain number. Strains S27, HN2S27, S30, and HNS30 were constructed previously (see Table 1 for nomenclature) and substantiated the importance of the neck/stalk region in effectively binding Fn (21, 23). Moreover, strain S30 lost its ability to efficiently adhere to Fn while strain S27 did not, suggesting a crucial role for domains 27, 28, and/or 29 in Fn binding.

**TABLE 1:** Bacterial strains used in this study

Strain	Characteristics	Source
B. henselae Marseille		
Wild type	Isolate from a patient diagnosed with cat scratch disease in Marseille, France (CP072904)	17, 37
ΔBadA-T	B. henselae Marseille BadA-negative mutant with a TN <KAN-2> transposon integrated in *badA*; kanamycin resistant (10 μg/mL)	8, 49
ΔBadA-D	B. henselae Marseille with *badA* deleted via homologous recombination	17
ΔBadA-T/pS27	B. henselae ΔBadA-T containing pS27, previously referred to as B. henselae badA-/pF12	23
ΔBadA-T/pS28	B. henselae ΔBadA-T containing pS28	This study
ΔBadA-T/pS29	B. henselae ΔBadA-T containing pS29	This study
ΔBadA-T/pS30	B. henselae ΔBadA-T containing pS30, previously referred to as B. henselae badA-/pN23	23
ΔBadA-T/pHN2S27	B. henselae ΔBadA-T containing pHN2S27, previously referred to as B. henselae badA-/pHN2F12	23
ΔBadA-T/pHNS30	B. henselae ΔBadA-T containing pHNS30, previously referred to as B. henselae badA-/pHN23	21
ΔBadA-T/pD16S28	B. henselae ΔBadA-T containing pD16S28	This study
ΔBadA-T/pD19S28	B. henselae ΔBadA-T containing pD19S28	This study
ΔBadA-T/pD25S28	B. henselae ΔBadA-T containing pD25S28	This study
ΔBadA-T/pD27S29	B. henselae ΔBadA-T containing pD27S29	This study
E. coli DH5α	Used in cloning steps and for collecting plasmids	NEB

Consequently, strains S28 and S29 were designed *in silico*, synthesized according to the *badA* sequence (GenBank MK993576.1) of B. henselae Marseille (17), and cloned into vector pBBR1MCS-5 (Table 2). Finally, B. henselae ΔBadA-T was transformed with the resulting plasmids via electroporation. Initial ELISA readouts showed a major drop in Fn binding capacity for strain S28 compared with those for strain S27, implying the presence of a key Fn binding motif within domain 27 (data not shown). Nevertheless, BadA might require a critical length or minimal amount of certain BadA motif regions (cumulative adhesion) to efficiently bind Fn. To elucidate this question, more BadA mutants (D16S28, D19S28, D25S28, and D27S29) were created by combining different BadA neck/stalk domains with S28 or S29, with D25S28 and D27S29 functioning as a scaffold (Fig. 3A). These domains (highlighted red in Fig. 2A) were selected to represent different variants of neck/stalk domains in comparison with domain 27 (Fig. 1 and 2). Domain 16 represents the most common group of “green-colored” domains, includes an FGG motif sequence (type 1), and is only 9 aa residues shorter than domain 27. Domain 19 lacks an FGG motif sequence and is considerably shorter but shows the highest pairwise amino acid sequence similarity with domain 27 (Fig. 2B) due to a highly similar coiled-coil region and DALL-neck tandem connector sequence. Domain 25 is likewise shorter and contains a slightly different coiled-coil and DALL-neck tandem connector sequence. Like for strains S28 and S29, all mutant sequences were synthesized, cloned into vector pBBR1MCS-5, and finally used to transform B. henselae ΔBadA-T.

**TABLE 2:** Plasmids used in this study

Plasmid	Characteristics	Source
pMK-RQ	GeneArt cloning vector, kanamycin resistance gene (50 μg/mL)	GeneArt
pMA	GeneArt cloning vector, ampicillin resistance gene (50 μg/mL)	GeneArt
pMS-RQ	GeneArt cloning vector, spectinomycin resistance gene (30 μg/mL)	GeneArt
pBBR1MCS-5	Broad host range vector, gentamycin resistance gene (10 μg/mL)	50
pS27	pBBR1MCS-5 containing a ca. 3.0-kb *badA* fragment (BadA S27), previously referred to as BadA F12	23
pS28	pBBR1MCS-5 containing a ca. 2.6-kb *badA* fragment (BadA S28)	This study
pS29	pBBR1MCS-5 containing a ca. 2.2-kb *badA* fragment (BadA S29)	This study
pS30	pBBR1MCS-5 containing a ca. 1.9-kb *badA* fragment (BadA S30), previously referred to as BadA N23	23
pHN2S27	pBBR1MCS-5 containing a ca. 4.3-kb *badA* fragment (BadA HN2S27), previously referred to as BadA HN2F12	23
pHNS30	pBBR1MCS-5 containing a ca. 3.1-kb *badA* fragment (BadA HNS30), previously referred to as BadA HN23	21
pD16S28	pBBR1MCS-5 containing a ca. 3.0-kb *badA* fragment (BadA D16S28)	This study
pD19S28	pBBR1MCS-5 containing a ca. 2.8-kb *badA* fragment (BadA D19S28)	This study
pD25S28	pBBR1MCS-5 containing a ca. 2.8-kb *badA* fragment (BadA D28S28)	This study
pD27S29	pBBR1MCS-5 containing a ca. 2.6-kb *badA* fragment (BadA D27S29)	This study

Expression of the truncated BadA mutants was verified via Western blotting, clearly demonstrating differences in molecular weight (MW) and corresponding with their respective estimated (trimeric) protein size (Fig. 3A and B). Accordingly, strains HN2S27 and S30 display the largest (estimated to be 343 kDa) and smallest (estimated to be 92 kDa) BadA mutant proteins, respectively. Nevertheless, TAAs are extremely stable proteins and largely remain as trimers in a sodium dodecyl sulfate-polyacrylamide gel electrophoresis (SDS-PAGE) gel, even after boiling the samples for 10 min (30). Strains ΔBadA-T and ΔBadA-D (*badA* deletion mutant) function as a negative control and do not show any BadA protein expression.

**FIG 4:**
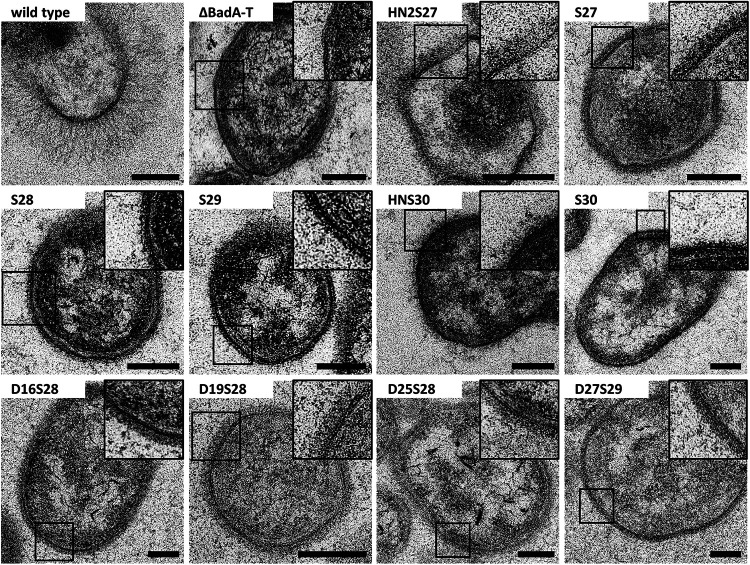
Analysis of bacterial surface expression of truncated and modified BadA proteins via transmission electron microscopy. TEM images of the B. henselae Marseille strains depict a dense layer of fibers attached to the outer membrane. In contrast, negative-control strain B. henselae Marseille ΔBadA-T displays a smooth outer membrane. BadA fiber lengths vary from ca. 20 nm (for BadA S30) to ca. 240 nm (for BadA wild type). Enlarged images of the BadA fiber structures are given in the framed boxes, respectively. Scale bars: 200 nm.

Proper expression and bacterial surface localization were verified via confocal laser scanning microscopy (CLSM) using anti-BadA IgG antibodies and transmission electron microscopy (TEM). BadA and all truncated mutants are present on the bacterial surface as is demonstrated by the green halo surrounding the 4′,6-diamidino-2-phenylindole (DAPI)-stained (intracellular) DNA (Fig. 3C). Negative-control strains B. henselae ΔBadA-T and ΔBadA-D do not display such a green halo. In addition, TEM images clearly show the membrane localization of expressed BadA mutants along the entire bacterial surface (Fig. 4). The B. henselae wild type displays a dense layer of long (>240 nm) BadA fibers, while the negative-control B. henselae ΔBadA-T has a smooth outer membrane. All other complemented B. henselae ΔBadA-T mutant strains display a strongly truncated BadA protein, present as short fibers on the bacterial surface varying in length from ca. 17 nm for strain S30 to ca. 45 nm for strain HN2S27.

### BadA-fibronectin binding is mediated mainly via neck/stalk residue motifs present in domains 19 and 27.

**FIG 5:**
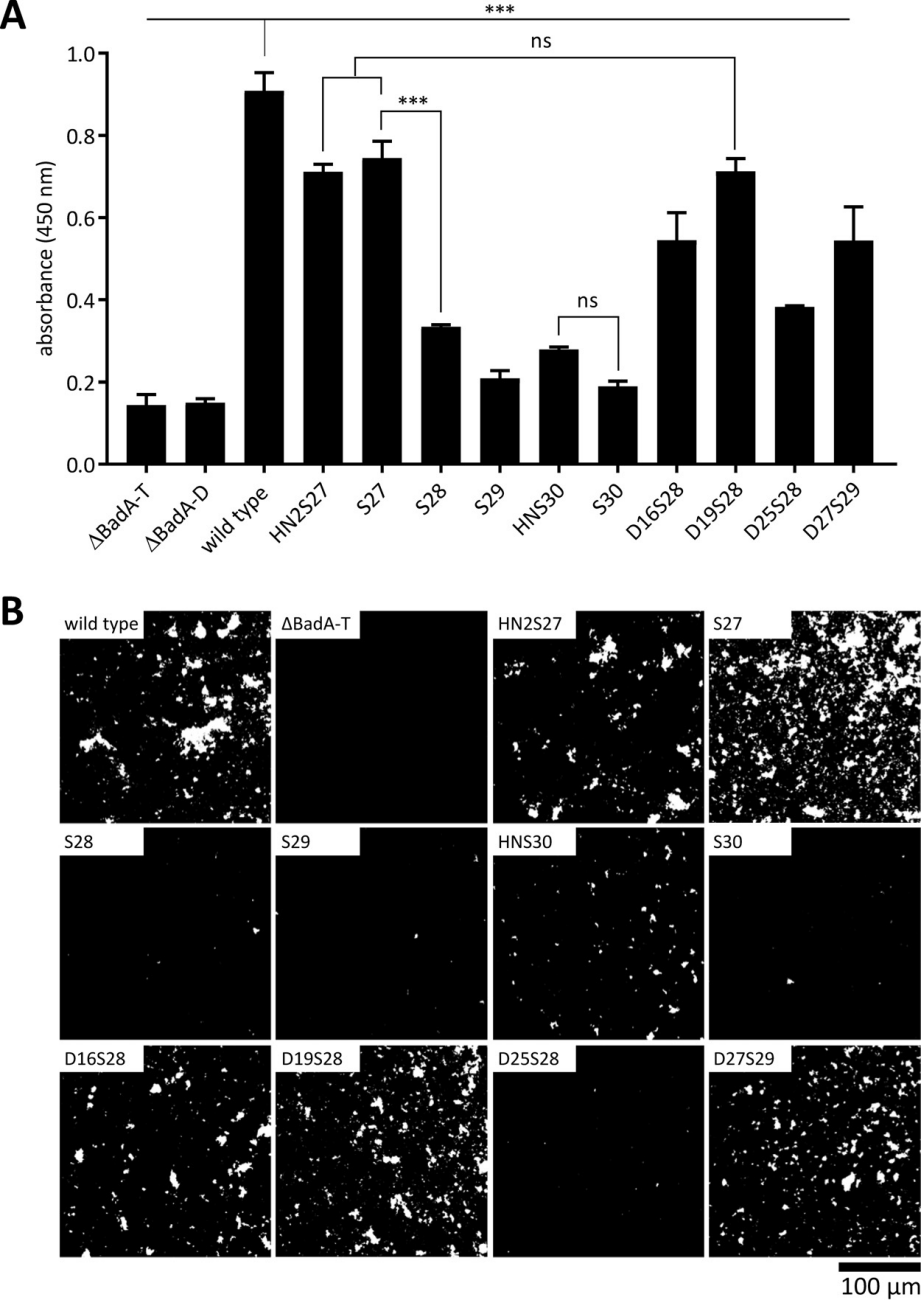
Analysis of the fibronectin binding capacity of the truncated and modified BadA mutants in B. henselae Marseille ΔBadA-T via ELISA and fluorescence microscopy. (A) The ability of BadA and truncated BadA constructs to bind immobilized human plasma Fn was quantified via whole-cell ELISA using anti-B. henselae IgG antibodies. All B. henselae Marseille mutant strains, including both negative-control strains ΔBadA-T (0.144) and ΔBadA-D (0.150), show a significant lower Fn binding capacity (***, *P < *0.001) than the wild-type strain (0.908). Similarly, strains S28 (0.334), S29 (0.209), HNS30 (0.279), S30 (0.189), and D25S28 (0.383) demonstrate a low binding to Fn. Both strains D16S28 (0.545) and D27S29 (0.544) show a significant increase in Fn binding compared with strain S28 and S29. No significant (ns) difference in Fn binding capacity is observed between strains D19S28 (0.713) and S27 (0.745). Furthermore, strains HNS30 and HN2S27 (0.711) do not bind Fn significantly stronger than their headless variants S30 and S27, respectively. (B) DAPI-stained fluorescence microscopy images visually confirm the above-given ELISA readouts by a higher number of Fn-attached bacteria (white). Scale bar: 100 μm.

Bacterial binding to Fn was analyzed via whole-cell ELISA (using anti-B. henselae IgG antibodies) and Fn-coated plates. All B. henselae mutant strains, including both negative-control strains ΔBadA-T and ΔBadA-D, displayed a significantly lower Fn binding strength (***, *P < *0.001) than the wild-type strain (Fig. 5A).

Despite the deletion of ca. 90% of the wild-type BadA protein sequence, strains HN2S27, S27, and D19S28 show a remarkably high Fn binding, close to the level of the wild-type strain. In contrast, strain S28 loses nearly its complete ability to bind Fn, nearing the level of both negative controls. A similar observation is made for strains S29 and S30 that display an even more truncated BadA protein. Compared with strain S28, strain D16S28 and certainly strain D19S28 show a significant increase in Fn binding, while D25S28 does not. Furthermore, strain D27S29 significantly exceeds the Fn binding ability of strain S29 and even strain S28 (Fig. 5A). In addition, no difference in Fn binding capacity is observed between strains HNS30 and HN2S27, and their respective headless variants S30 and S27, verifying that solely the neck/stalk region is crucial for strong Fn binding despite the major role of the head domain in numerous other biological functions (21, 23).

All ELISA results are supported by DAPI-stained fluorescence microscopy images. Accordingly, a higher adherence is observed visually for the wild-type strain and strains HN2S27, S27, D16S28, D19S28, and D27S29 (Fig. 5B). Finally, quantitative PCR (qPCR) results confirmed an approximate equal seeding number of bacterial cells for all strains used in the ELISA setup (see Fig. S2 in the supplemental material), as described in Materials and Methods.

### Polyclonal antibodies targeting the DALL motif sequence reduce Fn binding.

**FIG 6:**
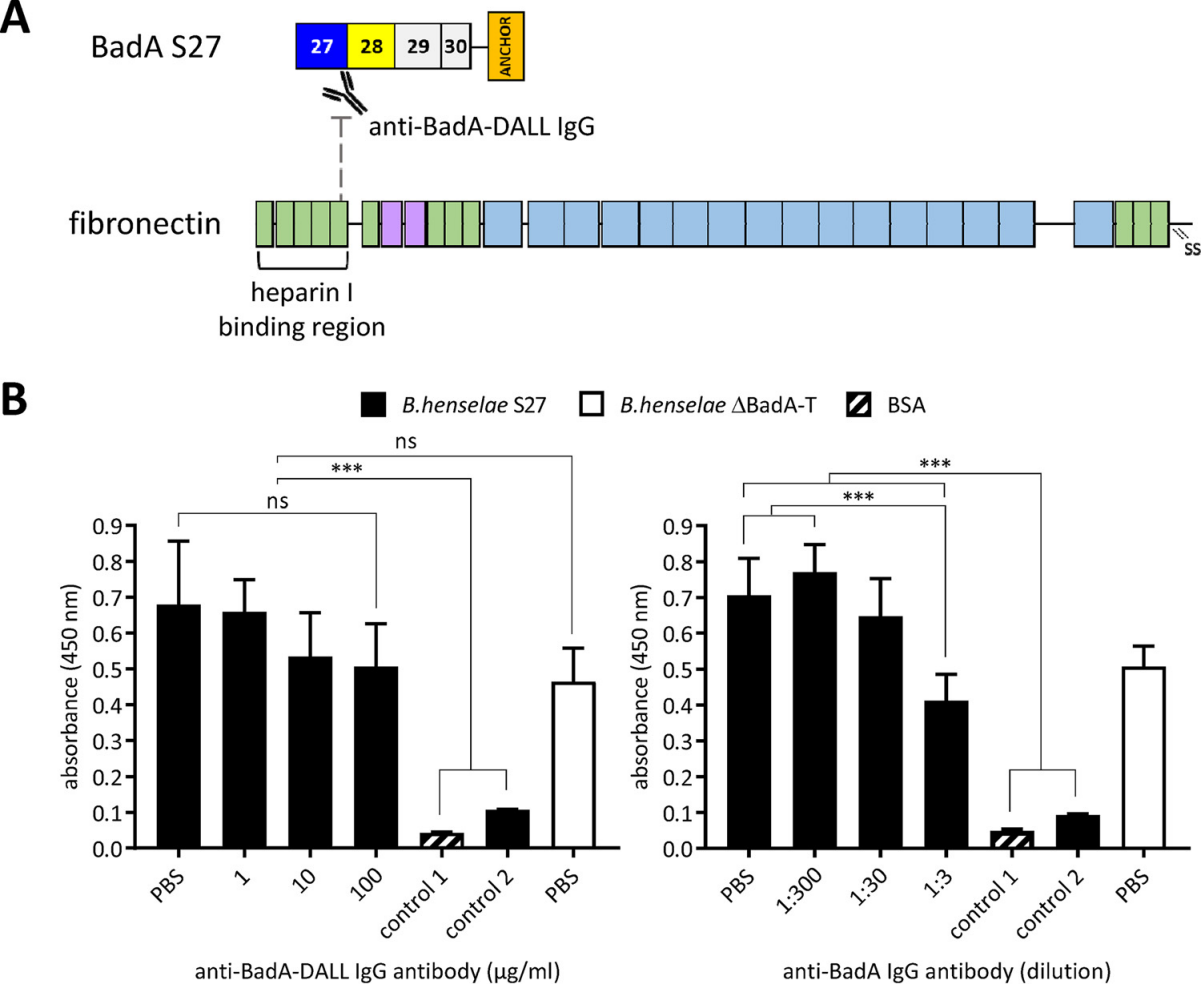
Analysis of the inhibiting effect on the Fn binding of B. henselae S27 using polyclonal antibodies targeting the DALL motif sequence within domain 27. (A) A reversed ELISA setup was used in which plates were first coated with either strains S27 or ΔBadA-T. The anti-BadA-DALL IgG antibodies target a 15-mer peptide (RHEKSKLEKGASKAI) present in the DALL motif sequence of the neck/stalk domain 27, of which a part was suggested to adhere the heparin I binding region in Fn (9). Bound human plasma Fn was identified via mouse IgG anti-Fn antibodies. (B) Inhibition of bacterial adherence of strain S27 to Fn, back to the level of strain ΔBadA-T (negative control), was observed when administering stepwise increasing concentrations of anti-BadA-DALL IgG antibodies (from 0 μg/mL to 100 μg/mL, respectively). Similar observations were made for anti-BadA IgG antibodies (positive control). Negative control 1 did not include the addition of bacteria, while negative control 2 included strain S27 without the addition of Fn. Negative controls 1 and 2 showed a significantly lower Fn binding (***, *P < *0.001).

Anti-BadA-DALL IgG antibodies were generated targeting a 15-mer peptide (RHEKSKLEKGASKAI) located within the DALL motif sequence of the neck/stalk domain 27 (Fig. 6A). This particular region was identified previously to be part of a BadA-Fn interaction site (9). The specificity of the anti-BadA-DALL IgG antibodies was confirmed via Western blotting, ELISA, and fluorescence microscopy (Fig. S3). Strain D19S28 contains a comparable sequence (RHEKSKLEKGVSKAT) and likewise reacted with the anti-BadA-DALL IgG antibodies via ELISA, while negative-control strains B. henselae Marseille ΔBadA-T, S28, S29, D16S28, and D25S28 did not react.

To check if the anti-BadA-DALL IgG antibodies might inhibit the binding of strain S27 to Fn, a reversed ELISA setup was used. Plates were coated with strain S27 and ΔBadA-T, after which they were incubated consecutively with anti-BadA-DALL IgG antibodies and human plasma Fn. By a stepwise increase of the concentration of anti-BadA-DALL IgG antibodies (from 1 μg/mL to 100 μg/mL), a corresponding decrease of Fn adhesion (Fig. 6B) was observed, similar to the level of strain ΔBadA-T, demonstrating that this 15-mer motif sequence within BadA is involved directly in Fn adherence.

### Length of truncated BadA constructs correlates with the immunoreactivity of human patient sera.

**FIG 7:**
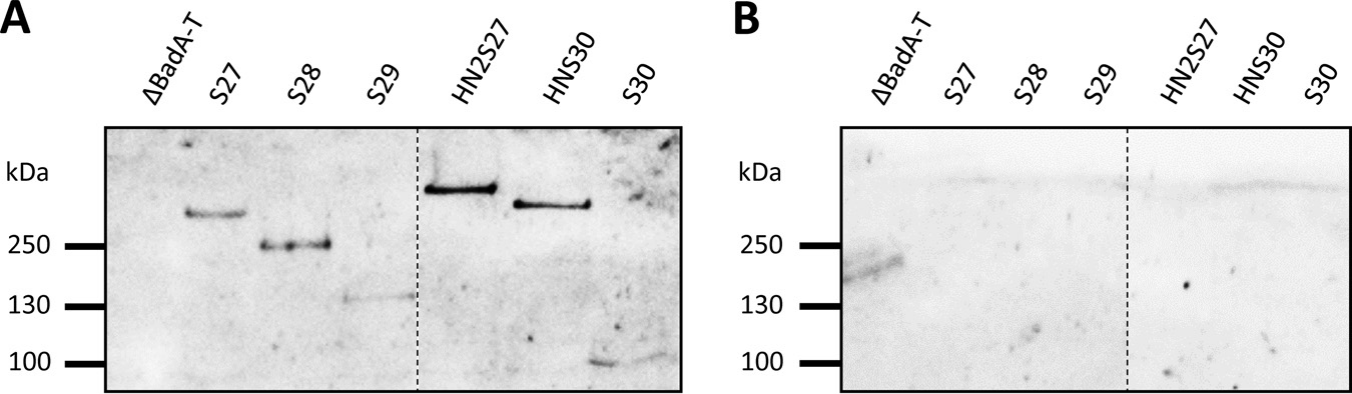
Representative Western blotting membranes demonstrating the immunoreactivity of truncated BadA constructs with human patient sera. (A) B. henselae BadA mutant strains were processed on an 8% SDS-PAGE gel. A representative Western blotting membrane with patient serum number 7 (B. henselae IgG titer of 5,120) is shown. BadA constructs with a head domain (HN2S27 and HNS30) show a more intense blotting band than those without (same number of bacteria loaded). The correct MW of all bands is given in Fig. 3B. (B) By using negative-control patient serum 14, no reacting bands are observed. All bacterial samples were each processed on the same nitrocellulose membrane but rearranged *in silico* (dotted lines).

BadA is an immunodominant protein (8, 22); however, the exact antigenic domains are unknown. The immunogenicity of truncated BadA proteins was assessed via Western blotting using human sera of 12 patients diagnosed with cat scratch disease, lymphadenopathy, or suspected endocarditis. B. henselae immunofluorescence assay (IFA) IgG titers for all serum samples ranged from 640 to 20,180 (see Table S2 in the supplemental material). Negative controls include two patient serum samples (IFA IgG titer, <80) and the B. henselae Marseille ΔBadA-T. Strains HN2S27, S27, and HNS30 display the largest BadA constructs and reacted with all 12 patient serum samples. The shorter BadA constructs S28 and S29 were observed only 9 and 8 times, respectively (Table 3). Larger BadA constructs might result in more potential antibody targets. The number of reactive bands for strain S30 (4 times), in comparison with strain HNS30 (12 times), demonstrates that the head domain is a major immunodominant region of BadA, which was also confirmed by stronger reactive blotting bands (Fig. 7).

**TABLE 3:** Seroreactivity of truncated BadA constructs in Western blotting membranes using patient serum samples^*a*^

Patient serum	Seroreactivity per BadA-truncated mutant^*b*^						
	ΔBadA-T	HN2S27	S27	S28	S29	HNS30	S30
1							
2							
3							
4							
5							
6							
7							
8							
9							
10							
11							
12							
13							
14							

a All tested B. henselae BadA mutant strains are listed. Each gray-filled box indicates a reacting band for the respective patient serum. Patient serum numbers 13 and 14 are negative controls (anti-B. henselae IgG titers < 80).

b The total number of observed reactive bands per truncated BadA construct for each mutant was 0, 12, 12, 9, 8, 12, and 4, respectively.

## DISCUSSION

Early-passage B. henselae isolates are characterized by extensive expression of the enormous outer membrane protein BadA that mediates adhesion to host cells and ECM proteins, including Fn (7, 8). The initial adhesion of B. henselae to Fn via BadA is essential in the early stages of host cell infection (9). B. henselae is subject to frequent genomic rearrangements via recombination events that contribute to host immune evasion and adaptation to differing host environments, resulting in highly variable *badA* gene variants and *badA* pseudogenes (17). The BadA neck/stalk region displays a typical domain architecture that contains conformationally predicted coiled-coil segments, FGG, DALL, and neck motif sequences (19, 20, 31). In this study, we generated numerous truncated and modified BadA constructs (Fig. 3A) and analyzed their ability to bind immobilized human plasma Fn to narrow down the number of potential Fn binding motif(s).

The low but measurable “background” Fn binding of negative-control strains ΔBadA-T and ΔBadA-D can be attributed to the B. henselae proteins Pap31, Omp89, and Omp43 (9, 32, 33). A weaker CLSM-fluorescence signal for shorter BadA constructs (strains S29 and S30) is due mainly to the smaller amount of potential binding targets for the anti-BadA IgG antibodies (Fig. 3C). Accordingly, a weaker number of reacting samples was detected when analyzing human sera for immunoreactivity with shorter BadA constructs. The short-chain lipopolysaccharide of B. henselae (34) and other outer membrane proteins should not obstruct binding in the case of the large wild-type BadA; however, it is unknown whether the shortest BadA constructs S29 (ca. 23 nm) and S30 (ca. 17 nm) are large enough to stick out from the glycolipid layer. Further analyses using an lipopolysaccharide (LPS)-deficient B. henselae strain might clarify this matter. Nevertheless, the TAA of Y. enterocolitica (*Yersinia* adhesin A) likewise measures only 23 nm and adheres to various ECM proteins, although to a lower degree than the B. henselae wild type (2, 7).

The deletion of 26 neck/stalk domains, resulting in the truncated BadA S27, proved to have only a little effect on Fn binding compared with the wild-type BadA. To further elucidate the motif sequence(s) mediating Fn binding, additional BadA mutants were constructed and expressed on the outer membrane of B. henselae ΔBadA-T. Consequently, and based on (i) the significant difference in ELISA binding between strains S27 and S28, (ii) the recovered and high Fn binding ability of strain D19S28, and (iii) the substantial increase in Fn adherence of strain D27S29 compared with that of strains S29 and S28, we suggest that Fn binding is mediated primarily by a common motif sequence present within the conserved DALL-neck tandem connector of domains 19 and 27. Moreover, domains 6 and 10 are highly similar to domain 27, while domains 15 and 23 are highly similar to domain 19. BadA adheres to both cellular and plasma Fn via specific interaction sites (9). Because of their different conformations, it was suggested that BadA binds regions in cellular Fn that are not accessible in plasma Fn. In that same study, only cellular Fn showed an interaction site with domain 27. However, a significant increase in our binding assay is observed between strains S29 and D27S29, demonstrating clearly that domain 27 also plays a role in the adherence to plasma Fn.

A complex network showing close interactions between BadA and Fn has been resolved previously using XL-MS (9), emphasizing the numerous repetitions and domain similarities seen throughout the BadA neck/stalk region. Six interaction sites were verified earlier by a competition-based ELISA using heparin and wild-type B. henselae (highlighted light blue in Fig. 2A; listed in Table S3 in the supplemental material) to effectively bind Fn *in vitro*. Moreover, four of those interaction sites are located exclusively in domains 19 and 27 and/or in highly similar domains. One of those BadA-Fn interaction sites (VNNNVTNKFNELTQSITNVTQQVK) is located within the coiled-coil motif of domain 19 and shows high similarity to the coiled-coil motif in domain 27. Three other identified peptides can be linked together (LEKGASKATQENSKITYLLDGDVSK) and appear within the DALL-neck tandem connector motif of domains 27 and 19. These XL-MS data are confirmed by the ELISA results presented here and emphasize the substantial role in Fn binding of both residue sequences within BadA domains 19 and 27 (and highly similar domains).

Targeting a specific region within the DALL motif of domain 27 (RHEKSKLEKGASKAI; partly present in the latter Fn-BadA interaction site) with increasing anti-BadA-DALL IgG antibody concentrations reduced the Fn binding ability of strain S27 in a dose-dependent manner. Therefore, these residues, indicated by arrowheads in Fig. 3A, are demonstrated to be involved directly in Fn adherence. The DALL-neck tandem connector is predicted to make BadA slightly bendable for a more efficient adhesion process. Moreover, hairpin β-strands are considered optimal interaction sites to adhere to the numerous unpaired β-strands present in Fn (29, 35). Comparable Fn binding mechanisms in Staphylococcus aureus and Streptococcus pyogenes are observed by forming extended tandem β-zippers (36).

The observed increase in Fn binding of strain D16S28, compared with strain S28, might be attributed to the presence of an FGG motif in domain 16. FGG motifs are distributed abundantly among the BadA neck/stalk region, are usually characterized by nonhelical structures that extend from the central axis of the TAA, and have been described previously as a potential Fn binding region (23). In addition, domain 16 and domain 28 also contain a previously identified BadA-Fn interaction site (9) within their FGG motif sequence, taking account of a single amino acid difference (TVNGEGKEEEK compared with TVNGEGKEEEQ). However, the Fn binding ability of a single FGG motif must be low as similar motif sequences present in domains 28 and 29 do not result in a strong Fn binding.

Because of the highly repetitive nature of the BadA neck/stalk region, one might speculate that the cumulation of Fn binding motifs leads to a linear increase of the overall Fn binding capacity. However, our data indicate that an increasing number of Fn-binding domains only slightly enhances the Fn binding capacity, as is demonstrated by the minimal difference in Fn binding between the wild-type strain (30 neck/stalk domains) and S27 (4 neck/stalk domains). Furthermore, earlier studies have shown that B. henselae strains HNS30 and HN2S27, each expressing a drastically truncated BadA protein (connected to a head domain), do not demonstrate differences in angiogenic reprogramming of infected host cells nor in their adherence to ECM proteins, with the exception of Fn (23). Therefore, the enormous length and repeated domains in the BadA neck/stalk region might primarily act as a “spacer” to facilitate adhesion and function as an “evolutionary toolbox” to mediate the generation of *badA* variations (17).

In conclusion, we demonstrated that specific repeated binding regions are necessary to reach a maximum Fn binding capacity, which in turn rapidly decreases upon removing certain domains that include important binding motifs (e.g., the DALL-neck connector within domains 19 and 27). In addition, inhibitory effects on the Fn adherence were observed when administering antibodies targeting a specific region in the DALL motif sequence of domain 27. We suggest that BadA adheres to Fn in a cumulative effort with quick saturation via unpaired β-sheet hairpins appearing in DALL-neck tandem motifs present within domains 19 and 27 (and highly similar domains). Future research should be focused on the production of recombinant BadA mutant proteins or single and trimeric neck/stalk motifs to further elucidate BadA-Fn binding mechanisms. The exact determination of specific BadA-Fn binding regions provides a basis toward developing antiadhesive compounds that might prevent the initial adherence of B. henselae and other TAA-expressing pathogens in the course of infection.

## MATERIALS AND METHODS

### Bacterial strains and culture conditions.

B. henselae Marseille (GenBank accession number CP072904) is a human isolate derived from a patient that was diagnosed with cat scratch disease (37). The Marseille wild-type strain functioned as a template for all cloning experiments, while the B. henselae Marseille ΔBadA-T strain (*badA* transposon mutant) (8) was used to express the truncated BadA mutants generated in this study (Table 1). B. henselae was cultured either in *Bartonella* liquid (BALI) medium supplemented with 10% Fn-depleted sterile fetal calf serum (FCS; Sigma-Aldrich, Deisenhofen, Germany) while shaking (120 RPM) for 3 days (38) or on Columbia blood agar (CBA) plates containing 5% sheep blood (Becton, Dickinson, Heidelberg, Germany); both cultures were placed in a humidified atmosphere at 37°C and with 5% CO_2_. Fully grown CBA plates and single colonies were obtained after 4 and 14 days of incubation, respectively. Escherichia coli DH5α competent bacteria (New England BioLabs [NEB], Frankfurt, Germany) were used for cloning experiments and were grown overnight (o/n) at 37°C on Luria/Miller (LB) agar plates (Carl Roth, Karlsruhe, Germany) or shaking (180 rpm) in LB broth. Selection markers kanamycin (MP Biomedicals, Eschwege, Germany), gentamicin (Gibco; Thermo Fisher Scientific, Darmstadt, Germany), and ampicillin (Carl Roth) were used at a final concentration of 30 μg/mL, 10 μg/mL, and 100 μg/mL, respectively.

Fn-depleted FCS was prepared as described previously (9). In short, gelatin Sepharose-4B (GE Healthcare, Munich, Germany) was added to heat-inactivated FCS and incubated o/n at 4°C. Gelatin Sepharose was removed via polypropylene columns (Bio-Rad, Dreieich, Germany), samples were subsequently filter sterilized, and aliquots were stored at −20°C.

### Construction and cloning of truncated BadA mutants.

To generate BadA mutant B. henselae Marseille strains (Table 1), fragments containing truncated and modified *badA* sequences were designed via SnapGene software (Insightful Science, San Diego, USA) (see Fig. S1 in the supplemental material), synthesized, and inserted into different vectors (i.e., pMK-RQ, pMS-RQ, and pMA) (Table 2) by GeneArt technology (Invitrogen; Thermo Fisher Scientific). The native promoter of B. henselae Marseille was suggested to be located in a ca. 250-bp region directly upstream of the *badA* start codon (nucleotide position of 1,362,098) and is included in all synthesized inserts (18, 39). The signal sequence contains 47 aa, is included in all synthesized BadA constructs, and is cleaved during transport into the periplasm (Fig. 1).

Synthesized fragments were amplified from their respective GeneArt plasmids (using primers S28domains_Fw and S28domains_Rv; see Table S1 in the supplemental material) and ligated immediately into the broad host range vector pBBR1MCS-5 (using primers pBBR1MCS-5_Fw and pBBR1MCS-5_Rv) via Gibson Assembly technology (NEB) (40). The resulting plasmids were propagated in heat shock-transformed E. coli DH5α and were later used for electroporation of electrocompetent B. henselae Marseille ΔBadA-T as described previously (17). In short, bacteria (40 μL; ca. 1 × 10^10^ cells/mL) were incubated on ice for 15 min together with 10 μg of purified plasmid DNA and 1 μL of TypeOne restriction inhibitor (Lucigen, Middleton, USA) in 0.2-cm-gap precooled electroporation cuvettes (Bio-Rad, Germany). Immediately after electroporation at 2.5 kV, 200 Ω, and 25 μF, samples were incubated for ca. 4 h in a humidified atmosphere at 37°C with 5% CO_2_ while gently shaking (120 rpm) in 1 mL of room temperature (RT) recovery broth (1% HEPES buffer [Sigma-Aldrich], 1% sodium pyruvate [Sigma-Aldrich], 5% FCS, and 5% rabbit blood lysate [Acila AG, Mörfelden-Walldorf, Germany] in RPMI 1640 medium with glutamine [PAN-Biotech, Aidenbach, Germany]). Transformed B. henselae ΔBadA-T was incubated subsequently for positive selection on kanamycin- and gentamicin-supplemented CBA plates.

After each transformation, the correct plasmid integration was checked via colony PCR using primers pBBR1MCS-5_GA_Fw and pBBR1MCS-5_GA_Rv. Insert sequences were verified to be error free via Sanger sequencing (data not shown; Microsynth Seqlab, Göttingen, Germany) using the same primers for colony PCR, as well as the primers S28domains_Fw, S28domains_Rv, BadA1_Fw, BadA2_Fw, and BadA3_Fw (Table S1). The expression of the various truncated BadA mutants was analyzed subsequently via immunofluorescence confocal laser scanning microscopy (CLSM) and transmission electron microscopy (TEM).

The differences in the protein size of the truncated and modified BadA mutants was analyzed via Western blotting using rabbit anti-BadA IgG antibodies (17). Briefly, bacterial samples were prepared by incubation in Laemmli sample buffer (Sigma-Aldrich) for 10 min at 95°C, separated via sodium dodecyl sulfate-polyacrylamide gel electrophoresis (SDS-PAGE), and transferred to a nitrocellulose membrane for 30 min at 25V in Towbin transfer buffer (with 20% glycerol). An o/n incubation at 4°C with rabbit anti-BadA IgG antibodies (1:4,000) was followed by a 90-min incubation at RT with horseradish peroxidase (HRP)-conjugated swine anti-rabbit IgG antibodies (1:2,000; Agilent-Dako, Carpinteria, USA). Processed membranes were developed using SuperSignal West Pico PLUS chemiluminescent substrate (Thermo Fisher Scientific) and analyzed on a ChemiDOC XRS+ system (Bio-Rad) with ImageLab V6.0.1. software (Bio-Rad).

### Detection and visualization of surface-expressed BadA proteins via immunofluorescence confocal laser scanning microscopy and transmission electron microscopy.

The surface expression of B. henselae truncated and modified BadA mutants was detected via CLSM using rabbit anti-BadA IgG antibodies (1:400) and visualized using TEM.

CLSM samples were processed as described previously (17). Briefly, BALI medium-grown bacteria were washed in phosphate-buffered saline (PBS; pH 7.2), air dried on microscopy slides (Knittel StarFrost, Braunschweig, Germany), and subsequently fixed using 3.75% paraformaldehyde (Electron Microscopy Sciences, Hatfield, USA). Bacterial-bound anti-BadA IgG antibodies were targeted with goat IgG anti-rabbit IgG conjugated to Alexa 488 (1:200; Dianova, Hamburg, Germany), and bacterial DNA was stained with 4′,6-diamidino-2-phenylindole (DAPI; 1 μg/mL; Sigma-Aldrich; Merck). Samples were mounted with fluorescence medium (Agilent-Dako) and analyzed on a Stellaris 8 confocal microscope (Leica, Mannheim, Germany) using Las X software (v4.4.0). Samples were captured (65% gain) with a 93× objective (glycerol) at an excitation and emission wavelength of 499 nm and 530 to 575 nm, respectively. Depicted CLSM images are representative of >3 images of each slide from different areas, were adjusted *in silico* to a brightness of 50% and a contrast of 10%, and were selected from >20 representative fields using conventional immunofluorescence microscopy (data not shown).

Bacterial samples analyzed via TEM were grown in BALI medium, fixed with 4% formaldehyde and 2.5% glutaraldehyde (both Electron Microscopy Sciences) in 0.1 M phosphate buffer (pH 7.4) for 90 min at RT, and stored at 4°C. Bacterial preparation was done following an adapted protocol as described earlier (17, 41). In short, fixed bacterial samples were washed in phosphate buffer (1,000 × *g*), embedded in 12% melted (37°C) gelatin (Merck), and subsequently cut into small cubes (1 mm^3^). After a second fixation in 1% glutaraldehyde for 5 min at 4°C, cubes were dehydrated by gradually increasing dimethylformamide concentration from 30% (in H_2_O) for 30 min at 0°C to 100% for 1.5 h at −35°C and by infiltration of Lowicryl K4M at −35°C. Finally, samples were polymerized by UV, cut into ultrathin sections, stained with uranyl acetate and lead citrate, and analyzed with a Tecnai Spirit electron microscope (Thermo Fisher Scientific) operated at 120 kV.

### Enzyme-linked immunosorbent binding of truncated BadA-expressing B. henselae mutants to fibronectin.

The ability of the B. henselae truncated and modified BadA mutants to bind immobilized human plasma Fn was assessed using an enzyme-linked immunosorbent assay (ELISA) and evaluated via fluorescence microscopy of DAPI-stained Fn-bound bacteria.

Reactions were carried out in Nunc Maxisorp flat-bottom 96-well plates (Thermo Fisher Scientific) that were coated o/n at 4°C with 1 μg of human plasma Fn (F2006; Sigma-Aldrich) in PBS and blocked for 2 h at 37°C with 2% (wt/vol) bovine serum albumin (Sigma-Aldrich) in washing buffer containing 0.05% vol/vol Tween 20 (Carl Roth) in PBS. BALI medium-cultured B. henselae strains were washed three times in PBS, and ca. 2.5 × 10^7^ bacterial cells were added to the wells for 2 h at 37°C. Attached bacteria were identified via anti-B. henselae IgG antibodies (1:1,000) (42) and HRP-conjugated swine IgG anti-rabbit IgG antibodies (1:2,000; Agilent-Dako) or via DAPI staining (1:250). Colorimetric absorbance (450 nm) was measured on a microplate Sunrise-Basic reader (Tecan, Wiesbaden, Germany) using 3,3′,5,5′-tetramethylbenzidine liquid substrate (TMB; Sigma-Aldrich) and 1 M HCl. DAPI-stained bacteria were analyzed on an Eclipse Ti microscope (Nikon, Tokyo, Japan) using NIS-Elements BR software (v4.30.02). Unbound bacteria and antibodies were removed by three consecutive washes. During all incubation steps, a protective seal was used to avoid evaporation. Assays were done in triplicate, and negative controls included samples without (w/o) the addition of bacteria or w/o prior Fn coating.

In addition, real-time quantitative PCR (qPCR) verified the initial addition of approximately equal amounts of bacterial cells to the ELISA (Fig. S2). In short, bacterial numbers of gene copy equivalents were calculated via an internal standard using the primers glyA_Fw and glyA_Rv (Table S1) and a 120-bp fragment of the housekeeping gene *glyA* (serine hydroxymethyltransferase) cloned in the pCR2.1-TOPO vector according to manufacturer’s guidelines (Thermo Fisher Scientific) as described before (43).

### Generation of a peptide-based anti-BadA-DALL antibody targeting a 15-mer BadA region.

A 15-mer peptide sequence, homologous to a specific region within BadA (RHEKSKLEKGASKAI), was synthesized and used as the antigen (ca. 75 μg/injection) for the generation of a rabbit anti-BadA-DALL IgG antibody (Eurogentec, Liège, Belgium). Rabbit preimmune serum was used to verify antibody specificity via Western blotting (data not shown).

Potential adhesion-inhibiting features of anti-BadA-DALL IgG antibodies were analyzed via a reversed ELISA, following similar experimental settings as described above. Briefly, wells were coated initially o/n at 4°C with ca. 5 × 10^7^ bacterial cells (i.e., B. henselae S27 or ΔBadA-T) and subsequently blocked for 2h at 37°C with 2% (wt/vol) bovine serum albumin. Attached bacteria were incubated consecutively with a dilution series of anti-BadA-DALL IgG antibodies (i.e., 0 μg/mL, 1 μg/mL, 10 μg/mL, and 100 μg/mL, respectively) and with 1 μg of human plasma Fn in PBS, both for 1.5 h at 37°C. Anti-BadA IgG antibodies were similarly diluted and used as positive controls. Bound Fn was identified via mouse IgG anti-Fn antibodies (1:1,000; Becton, Dickinson) and HRP-conjugated goat IgG anti-mouse IgG antibodies (1:1,000; Agilent-Dako). Colorimetric absorbance (450 nm) was measured using TMB and 1 M HCl. Assays were done in quintuplicates, and negative controls included samples w/o the addition of bacteria (BSA) or Fn (PBS).

### BadA *in silico* analyses.

BadA is organized in 30 distinct domains according to their conserved neck motif sequence (Fig. 1). A further subdivision was made via the daTAA server that implements profile hidden Markov models and sequence homology-based deduction of knowledge to determine domain boundaries, while making use of the common modular TAA architecture (19). The presence of α-helixes, β-strands, and coiled-coil segments was assessed via Quick2D (44–46). A multiple protein sequence alignment of the numerous BadA domains was done via ClustalΩ (47) and adjusted manually according to physicochemical amino acid properties and daTAA server-defined sequence motifs (Fig. 2). A pairwise BadA domain similarity plot was created in a three-dimensional space using cluster analysis of sequences (CLANS) (Fig. 1A) (48). General gene analyses, *in silico* cloning steps, and the assessment of sequencing results were performed via SnapGene software (Insightful Science).

### Detection of truncated BadA constructs via Western blotting using human patient sera.

Human patient sera were used for Western blotting. This procedure was approved by the ethics committee of the University Hospital Frankfurt am Main (ethics proposal number 423/11).

The sera of patients were taken routinely by physicians or general practitioners for medical reasons to confirm or to exclude the clinical diagnosis of cat scratch disease or *Bartonella* infection and were sent to the German consiliary laboratory for *Bartonella* infections (Frankfurt, Germany; appointed by the Robert Koch-Institute, Berlin, Germany). Laboratory testing of the sera was performed under strict quality-controlled criteria (laboratory accreditation according to ISO 15189:2014 standards; certificate number D–ML–13102–01–00, valid through 25 January 2025) at the Institute for Medical Microbiology and Infection Control, University Hospital Frankfurt am Main, Germany. Indirect immunofluorescence assays (IFAs) were performed using the B. henselae/Bartonella quintana (IgG) kit (Euroimmun, Lübeck, Germany) with some modifications. A standard serum dilution series from 1:80 to 1:320 and higher was screened for anti-B. henselae IgG antibodies. The results were evaluated positive when specific fluorescence signals were detected at titers ≥320. Control sera were evaluated as IFA-negative at a titer of <80.

A selection of B. henselae truncated BadA mutants was processed via Western blotting (see above) using human patient sera (Fig. 7). In short, whole-cell bacterial samples were separated on an 8% SDS-PAGE gel and blotted subsequently on a nitrocellulose membrane. Resulting membranes were incubated consecutively with human patient serum (1:250) and with rabbit anti-human IgG antibodies (1:800; Agilent-Dako). B. henselae IgG titers for patient serum samples ranged from 640 to 20,180 (Table S2). Two negatively tested patient serum samples (IFA titer, <80) were used as negative controls.

### Statistical analysis.

Statistical analyses were performed on Prism v7.04 (GraphPad Software, San Diego, USA) using a one-way analysis of variance (ANOVA) and assuming parametric data distribution. A *P* value of <0.01 was considered statistically significant.
