# Supplementary material for: Adhesion of Bartonella henselae to Fibronectin Is Mediated via Repetitive Motifs Present in the Stalk of Bartonella Adhesin A
Source: Microbiol Spectr. 2022 Sep 27;10(5):e02117-22. doi: 10.1128/spectrum.02117-22 (PMC9602544; doi:10.1128/spectrum.02117-22)
Supplement: Supplemental file 1 — Fig. S1 to S3 and Tables S1 to S3. Download spectrum.02117-22-s0001.pdf, PDF file, 0.4 MB [file spectrum.02117-22-s0001.pdf]

-supplementary information-

Adhesion of *Bartonella henselae* to fibronectin is mediated via repetitive motifs present in the stalk of *Bartonella* adhesin A

Arno Thibau, Diana J. Vaca, Marlene Bagowski, Katharina Hipp,

Daniela Bender, Wibke Ballhorn, Dirk Linke, Volkhard A. J. Kempf

Table of contents

Supplementary Figures

Figure S1. Synthesised and truncated *badA* sequences used in this study. 2

Figure S2. Control of bacterial numbers inoculated in Fn binding assays. 12

Figure S3. Analysis of the specificity of anti-BadA-DALL IgG antibodies. 13

Supplementary Tables

Table S1. Primers used in this study. 14

Table S2. Human patient sera used in this study. 14

Table S3. Comparative overview of cross-linking mass spectrometry identified interaction sites between BadA and Fn. 15

Figure S1. Synthesised and truncated *badA* sequences used in this study. Each grey highlighted region translates to the truncated BadA amino acid sequence shown directly underneath. The underlined sequence represents the cleaved signal sequence. Purple highlighted nucleotides and amino acids depict the transition site of two combined *badA* regions.

(A) BadA HN2S27

gene sequence

CTGAATTTAGAGAGTGTAAGCTTTTATAGAAGCGTGCTGTTCTCTTTGAAAAGGAATGGTATTGTTCACAAAAAGTACTGTTTTTATTATGAAC
TAAAAAAATTTATTTTTAGCTTGCTATTTTACTCAATAGAGGATAGTGATACAGAAGGTATATCAGTATACTCATTTTAATTATAACTTCAAAA
GGGGAGGAAGTAATGCGTAAAAGACGAAACGCCACTCTAAAAGCAAATTTACATACCGCATCACACTCAATATAAAGAAACACTCGTAACAGAA
ATCAACTAAGCATACAGATTTCTTTTAAATATTCTTCAAATTCTCTTATTATTAAGAAAAGATGCTCCTTAATGAAAAAATTTTTTAATAAAAC
AGATAGCAATAAAAGAATGATTGAAATATTATTTAAACAACACCACCCTAACGTAAAACGTCTTAATATTTAAAACAGAAAAATTCTTTTTTAA
GTACACAACAAAAACAACCGCTCAACCCCTATTACAATCCAAATGCGCTATTTACACGCTTCCTACCAAGCTTTCGCATTCAGATTTCATTACA
GAAAGTACACACAAAATAAAAATAAAGACTCAAAACGTTCCCAATTTGACCACCCTCCTTATTTTAATCCTCATTACAAGGGAGTAGGTAATAC
TAAAATGTGTCTTTTTTATGTTTTGGATGTGCTTTGTAATTTTTTTCATTGGAGAATTTATT**ATG**AAAAAATTATCTGTCACATCAAAGAGACA
ATATAATTTATATGCTTCGCCTATTTCTCGACGTTTATCTTTGTTAATGAAGCTCTCATTGGAAACTGTAACAGTTATGTTCTTATTGGGTGCA
TCTCCTGTATTGGCTTCGAATCTTGCGCTTACAGGAGCAAAGAATCTGAGTCAAAACTCTCCAGGTGTAAATTACTCTAAAGGTAGCCATGGTA
GTATTGTTCTCTCTGGTGATGATGATTTTTGCGGTGCGGATTATGTTCTTGGTCGTGGAGGCAATTCTACTGTACGTAATGGGATTCCAATAAG
TGTAGAAGAAGAATATGAGAGATTTGTCAAACAAAAATTAATGAATAATGCTACTTCTCCTTATAGTCAGAGTTCAGAGCAACAAGTTTGGACT
GGTGATGGGCTAACAAGCAAAGGTTCGGGTTATATGGGAGGGAAGTCGACTGACGGTGATAAAAATATCTTGCCTGAGGCTTATGGTATATATT
CTTTTGCAACTGGTTGTGGTTCTTCTGCGCAGGGGAATTATTCAGTTGCATTTGGTGCAAATGCAACTGCACTTACTGGGGGGTCGCAAGCTTT
TGGTGTTGCTGCACTTGCAAGTGGAAGGGTAAGTGTTGCTATTGGTGTAGGGTCAGAAGCGACGGGAGAGGCTGGAGTTTCTTTGGGTGGACTC
TCAAAGGCAGCTGGTGCTCGTAGTGTTGCTATAGGGACGCGGGCTAAAGCTCAGGGTGAAGAATCTATTGCGATAGGTAGTAGCGTAAAGAATG
GTGATAAGGACGGTTCAGCTGTAGCGCAGGGTGCAAAAGCGATTGCTATAGGTTCTAATTCTATTAGTTTTCAGCACTATGCAGTTGCGGTTGG
TGCTAAAGCCCATGCTCTTCTCTCGAAAACTGTTGCCTTGGGTTATGATTCTGTTGCTGATGTTGATGCTGGCATTAGAGGTTATGATCCTGTG
GAGGATGAGCCATCGAAAGACGTTAGTTTTGTATGGAAAAGCTCTCTAGGTGCTGTTAGTGTTGGTAATCGTAAAGAAGGCTTAACGCGACAAA
TTATAGGAGTTGCAGCTGGTACTGAAGACACTGATGCAGTAAATGTTGCACAGCTAAAAGCATTAAGGGGAATGATATCAGAAAAAGGAGGTTG
GAATCTTACTGTTAATAATGACAATAATACAGTTGTTAGCTCAGGTGGTGCATTAGATTTGTCATCTGGAAGTAAAAATCTCAAAATTGTAAAA
GATGGAAAAAAGAATAATGTAACCTTTGATGTCGCTAGGGATCTCACGTTAAAGAGCATAAAATTAGACGGTGTTACTTTAAATGAAACAGGTT
TATTTATTGCAAACGGACCACAAATCACCGCTTCAGGTATTAATGCTGGTAGTCAAAAAATTACAGGCGTAGCAGAGGGTACTGATGCGAACGA
TGCAGTAAACTTTGGACAACTGCATACAATCGGTGAGGATGTTGCAAAATTCTTGGGTGGAGATGCAGCTTTTAAAGATGGCGCTTTTACCGGC
CCAACTTATAAGTTGTCGAATATTGATGCAAAGGGTGATGTACAACAGAGTGAGTTTAAAGATATAGGTTCAGCCTTTGCGGGTCTTGATACGA
ACATCAAGAATGTCAATAATAATGTAACGAATAAGCTCAGTGAACTTACTCAAAACATAACGACTGTTACGCAACAGGTAAAAGGCAATGCCTT
ATTATGGAGCGATGAAGCTAATGCCTTTGTGGCGCGTCATGAAAAGAGCAAGTTAGAAAAAGGTGCATCTAAAGCGATACAAGAAAACAGCAAG
ATTACGTATCTGTTAGATGGTGATGTTTCGAAAGGTTCCACGGATGCCGTTACTGGTGGTCAGCTTTATTCAATGAGCAATATGCTTGCGACCT
ATTTGGGTGGTAACGCTAAATATGAGAATGGTGAATGGACCGCACCTACCTTTAAGGTTAAAACAGTTAACGGTGAAGGCAAGGAAGAAGAGCA
AACTTATCAGAATGTAGCGGAAGCTTTGACTGGAGTTGGTACGTCTTTCACCAATATAAAAAGTGAGATTGCCAAACAGATTAATCATCTCCAG
TCTGATGATTCAGCGGTTATTCATTATGATAAGAATAAAGATGAAACTGGCACCATTAATTATGCGAGTGTAACTTTGGGTAAAGGTGAAGATT
CTGCAGCTGTTGCCCTTCATAATGTCGCTGCAGGTAATATTGCTAAGGATTCACGTGATGCAATCAATGGTTCTCAGCTTTATTCTTTGAACGA
GCAGTTATTGACCTATTTTGGCGGTGATGCTGGCTATAAAGATGGGCAATGGATAGCTCCCAAGTTCCATGTTTTGCAGTTCAAGAGTGATGGT
AGTTCTGGTGAGAAGGAGAGCTATGATAATGTAGCGGCTGCGTTTGAAGGAGTTAACAAAAGTCTTGCAGGTATGAACGAGCGTATTAATAATG
TTACTGCTGGCCAGAATGTTTCGTCGAGCAGTTTAAATTGGAATGAGACGGAGGGAGGTTATGACGCTCGTCATAATGGTGTGGACAGTAAGCT
TACGCATGTAGAGAATGGTGACGTATCCGAAAAATCGAAAGAAGCCGTTAATGGAAGTCAACTATGGAATACGAATGAGAAAGTTGAAGCGGTT
GAGAAGGATGTAAAGAATATTGAGAAGAAGGTACAAGATATTGCTACAGTAGCAGATAGTGCTGTTAAGTATGAGAAAGATAGTACTGGCAAGA
AAACGAATGTAATCAAATTAGTTGGTGGGAGTGAAAGTGAGCCAGTATTGATAGACAATGTAGCGGATGGTAAAATTGAAGCAGACTCTAAGCA
GGCAGTCAATGGAGGTCAGTTGCGTGATTATACTGAGAAACAGATGAAGATAGTGCTTGATGATGCGAAGAAATATACGGATGAACGCTTCAAT
GATGTCGTCAATAATGGTATTAATGAGGCTAAAGCTTATACAGATGTGAAGTTTGAGGCTTTAAGTTACACTGTTGAGGAAGTCCGGAAAGAAG
CAAGACAAGCAGCGGCTATTGGTTTAGCAGTATCTAACTTACGTTACTATGATATACCAGGATCTTTAAGTCTTTCATTTGGTACGGGTATATG
GCGTAGTCAGTCTGCATTTGCTATTGGTGCTGGTTATACATCTGAAGATGGCAATATTCGTTCTAATTTATCTATCACGAGTTCTGGTGGTCAG
TGGGGAGTAGGCGCAGGGATTACTTTGAGACTGAAA**TGA**TAAAAAAACTAATATTATGATAGAAAAACGAAGTATTTTGATAAATATTCTGTTC
TTCCTTGCCTTATTAGGCAAGGGAGAAAGTTTTGCTGATGAAAACGATAGTGTTTATACGGTGCATCCACCGCATTTATCTATTCCTAATGGGG
TAGCGGGTGAAACACGTCGAATCATCATGCAGTTTTATTATTGGACTTTAATTTGTGATGAAAAACAAAAGCTTAGGCAAGGCATATGTAATGT
GACGCAAACTGTCCATGATAAGGAAGGCAATACTATTTTCAGTTGGTCTCTTGTTTCTACGAAAAA

protein sequence

MKKLSVTSKRQYNLYASPISRRLSLLMKLSLETVTVMFLLGASPVLASNLALTGAKNLSQNSPGVNYSKGSHGSIVLSGDDDFCGADYVLGRGG
NSTVRNGIPISVEEEYERFVKQKLMNNATSPYSQSSEQQVWTGDGLTSKGSGYMGGKSTDGDKNILPEAYGIYSFATGCGSSAQGNYSVAFGAN
ATALTGGSQAFGVAALASGRVSVAIGVGSEATGEAGVSLGGLSKAAGARSVAIGTRAKAQGEESIAIGSSVKNGDKDGSAVAQGAKAIAIGSNS
ISFQHYAVAVGAKAHALLSKTVALGYDSVADVDAGIRGYDPVEDEPSKDVSFVWKSSLGAVSVGNRKEGLTRQIIGVAAGTEDTDAVNVAQLKA
LRGMISEKGGWNLTVNNDNNTVVSSGGALDLSSGSKNLKIVKDGKKNNVTFDVARDLTLKSIKLDGVTLNETGLFIANGPQITASGINAGSQKI
TGVAEGTDANDAVNFGQLHTIGEDVAKFLGGDAAFKDGAFTGPTYKLSNIDAKGDVQQSEFKDIGSAFAGLDTNIKNVNNNVTNKLSELTQNIT
TVTQQVKGNALLWSDEANAFVARHEKSKLEKGASKAIQENSKITYLLDGDVSKGSTDAVTGGQLYSMSNMLATYLGGNAKYENGEWTAPTFKVK
TVNGEGKEEEQTYQNVAEALTGVGTSFTNIKSEIAKQINHLQSDDSAVIHYDKNKDETGTINYASVTLGKGEDSAAVALHNVAAGNIAKDSRDA
INGSQLYSLNEQLLTYFGGDAGYKDGQWIAPKFHVLQFKSDGSSGEKESYDNVAAAFEGVNKSLAGMNERINNVTAGQNVSSSSLNWNETEGGY
DARHNGVDSKLTHVENGDVSEKSKEAVNGSQLWNTNEKVEAVEKDVKNIEKKVQDIATVADSAVKYEKDSTGKKTNVIKLVGGSESEPVLIDNV
ADGKIEADSKQAVNGGQLRDYTEKQMKIVLDDAKKYTDERFNDVVNNGINEAKAYTDVKFEALSYTVEEVRKEARQAAAIGLAVSNLRYYDIPG
SLSLSFGTGIWRSQSAFAIGAGYTSEDGNIRSNLSITSSGGQWGVGAGITLRLK-

(B) BadA S27

gene sequence

CTGAATTTAGAGAGTGTAAGCTTTTATAGAAGCGTGCTGTTCTCTTTGAAAAGGAATGGTATTGTTCACAAAAAGTACTGTTTTTATTATGAAC
TAAAAAAATTTATTTTTAGCTTGCTATTTTACTCAATAGAGGATAGTGATACAGAAGGTATATCAGTATACTCATTTTAATTATAACTTCAAAA
GGGGAGGAAGTAATGCGTAAAAGACGAAACGCCACTCTAAAAGCAAATTTACATACCGCATCACACTCAATATAAAGAAACACTCGTAACAGAA
ATCAACTAAGCATACAGATTTCTTTTAAATATTCTTCAAATTCTCTTATTATTAAGAAAAGATGCTCCTTAATGAAAAAATTTTTTAATAAAAC
AGATAGCAATAAAAGAATGATTGAAATATTATTTAAACAACACCACCCTAACGTAAAACGTCTTAATATTTAAAACAGAAAAATTCTTTTTTAA
GTACACAACAAAAACAACCGCTCAACCCCTATTACAATCCAAATGCGCTATTTACACGCTTCCTACCAAGCTTTCGCATTCAGATTTCATTACA
GAAAGTACACACAAAATAAAAATAAAGACTCAAAACGTTCCCAATTTGACCACCCTCCTTATTTTAATCCTCATTACAAGGGAGTAGGTAATAC
TAAAATGTGTCTTTTTTATGTTTTGGATGTGCTTTGTAATTTTTTTCATTGGAGAATTTATT**ATG**AAAAAATTATCTGTCACATCAAAGAGACA
ATATAATTTATATGCTTCGCCTATTTCTCGACGTTTATCTTTGTTAATGAAGCTCTCATTGGAAACTGTAACAGTTATGTTCTTATTGGGTGCA
TCTCCTGTATTGGCTTCGAATCTTGCGGGACAGATTCATACAATCGGTGAGGATGTTGCAAAATTCTTGGGTGGAGATGCAGCTTTTAAAGATG
GCGCTTTTACCGGCCCAACTTATAAGTTGTCGAATATTGATGCAAAGGGTGATGTACAACAGAGTGAGTTTAAAGATATAGGTTCAGCCTTTGC
GGGTCTTGATACGAACATCAAGAATGTCAATAATAATGTAACGAATAAGCTCAGTGAACTTACTCAAAACATAACGACTGTTACGCAACAGGTA
AAAGGCAATGCCTTATTATGGAGCGATGAAGCTAATGCCTTTGTGGCGCGTCATGAAAAGAGCAAGTTAGAAAAAGGTGCATCTAAAGCGATAC
AAGAAAACAGCAAGATTACGTATCTGTTAGATGGTGATGTTTCGAAAGGTTCCACGGATGCCGTTACTGGTGGTCAGCTTTATTCAATGAGCAA
TATGCTTGCGACCTATTTGGGTGGTAACGCTAAATATGAGAATGGTGAATGGACCGCACCTACCTTTAAGGTTAAAACAGTTAACGGTGAAGGC
AAGGAAGAAGAGCAAACTTATCAGAATGTAGCGGAAGCTTTGACTGGAGTTGGTACGTCTTTCACCAATATAAAAAGTGAGATTGCCAAACAGA
TTAATCATCTCCAGTCTGATGATTCAGCGGTTATTCATTATGATAAGAATAAAGATGAAACTGGCACCATTAATTATGCGAGTGTAACTTTGGG
TAAAGGTGAAGATTCTGCAGCTGTTGCCCTTCATAATGTCGCTGCAGGTAATATTGCTAAGGATTCACGTGATGCAATCAATGGTTCTCAGCTT
TATTCTTTGAACGAGCAGTTATTGACCTATTTTGGCGGTGATGCTGGCTATAAAGATGGGCAATGGATAGCTCCCAAGTTCCATGTTTTGCAGT
TCAAGAGTGATGGTAGTTCTGGTGAGAAGGAGAGCTATGATAATGTAGCGGCTGCGTTTGAAGGAGTTAACAAAAGTCTTGCAGGTATGAACGA
GCGTATTAATAATGTTACTGCTGGCCAGAATGTTTCGTCGAGCAGTTTAAATTGGAATGAGACGGAGGGAGGTTATGACGCTCGTCATAATGGT
GTGGACAGTAAGCTTACGCATGTAGAGAATGGTGACGTATCCGAAAAATCGAAAGAAGCCGTTAATGGAAGTCAACTATGGAATACGAATGAGA
AAGTTGAAGCGGTTGAGAAGGATGTAAAGAATATTGAGAAGAAGGTACAAGATATTGCTACAGTAGCAGATAGTGCTGTTAAGTATGAGAAAGA
TAGTACTGGCAAGAAAACGAATGTAATCAAATTAGTTGGTGGGAGTGAAAGTGAGCCAGTATTGATAGACAATGTAGCGGATGGTAAAATTGAA
GCAGACTCTAAGCAGGCAGTCAATGGAGGTCAGTTGCGTGATTATACTGAGAAACAGATGAAGATAGTGCTTGATGATGCGAAGAAATATACGG
ATGAACGCTTCAATGATGTCGTCAATAATGGTATTAATGAGGCTAAAGCTTATACAGATGTGAAGTTTGAGGCTTTAAGTTACACTGTTGAGGA
AGTCCGGAAAGAAGCAAGACAAGCAGCGGCTATTGGTTTAGCAGTATCTAACTTACGTTACTATGATATACCAGGATCTTTAAGTCTTTCATTT
GGTACGGGTATATGGCGTAGTCAGTCTGCATTTGCTATTGGTGCTGGTTATACATCTGAAGATGGCAATATTCGTTCTAATTTATCTATCACGA
GTTCTGGTGGTCAGTGGGGAGTAGGCGCAGGGATTACTTTGAGACTGAAA**TGA**TAAAAAAACTAATATTATGATAGAAAAACGAAGTATTTTGA
TAAATATTCTGTTCTTCCTTGCCTTATTAGGCAAGGGAGAAAGTTTTGCTGATGAAAACGATAGTGTTTATACGGTGCATCCACCGCATTTATC
TATTCCTAATGGGGTAGCGGGTGAAACACGTCGAATCATCATGCAGTTTTATTATTGGACTTTAATTTGTGATGAAAAACAAAAGCTTAGGCAA
GGCATATGTAATGTGACGCAAACTGTCCATGATAAGGAAGGCAATACTATTTTCAGTTGGTCTCTTGTTTCTACGAAAAA

protein sequence

MKKLSVTSKRQYNLYASPISRRLSLLMKLSLETVTVMFLLGASPVLASNLAGQIHTIGEDVAKFLGGDAAFKDGAFTGPTYKLSNIDAKGDVQQ
SEFKDIGSAFAGLDTNIKNVNNNVTNKLSELTQNITTVTQQVKGNALLWSDEANAFVARHEKSKLEKGASKAIQENSKITYLLDGDVSKGSTDA
VTGGQLYSMSNMLATYLGGNAKYENGEWTAPTFKVKTVNGEGKEEEQTYQNVAEALTGVGTSFTNIKSEIAKQINHLQSDDSAVIHYDKNKDET
GTINYASVTLGKGEDSAAVALHNVAAGNIAKDSRDAINGSQLYSLNEQLLTYFGGDAGYKDGQWIAPKFHVLQFKSDGSSGEKESYDNVAAAFE
GVNKSLAGMNERINNVTAGQNVSSSSLNWNETEGGYDARHNGVDSKLTHVENGDVSEKSKEAVNGSQLWNTNEKVEAVEKDVKNIEKKVQDIAT
VADSAVKYEKDSTGKKTNVIKLVGGSESEPVLIDNVADGKIEADSKQAVNGGQLRDYTEKQMKIVLDDAKKYTDERFNDVVNNGINEAKAYTDV
KFEALSYTVEEVRKEARQAAAIGLAVSNLRYYDIPGSLSLSFGTGIWRSQSAFAIGAGYTSEDGNIRSNLSITSSGGQWGVGAGITLRLK-

(C) BadA S28

gene sequence

CTGAATTTAGAGAGTGTAAGCTTTTATAGAAGCGTGCTGTTCTCTTTGAAAAGGAATGGTATTGTTCACAAAAAGTACTGTTTTTATTATGAAC
TAAAAAAATTTATTTTTAGCTTGCTATTTTACTCAATAGAGGATAGTGATACAGAAGGTATATCAGTATACTCATTTTAATTATAACTTCAAAA
GGGGAGGAAGTAATGCGTAAAAGACGAAACGCCACTCTAAAAGCAAATTTACATACCGCATCACACTCAATATAAAGAAACACTCGTAACAGAA
ATCAACTAAGCATACAGATTTCTTTTAAATATTCTTCAAATTCTCTTATTATTAAGAAAAGATGCTCCTTAATGAAAAAATTTTTTAATAAAAC
AGATAGCAATAAAAGAATGATTGAAATATTATTTAAACAACACCACCCTAACGTAAAACGTCTTAATATTTAAAACAGAAAAATTCTTTTTTAA
GTACACAACAAAAACAACCGCTCAACCCCTATTACAATCCAAATGCGCTATTTACACGCTTCCTACCAAGCTTTCGCATTCAGATTTCATTACA
GAAAGTACACACAAAATAAAAATAAAGACTCAAAACGTTCCCAATTTGACCACCCTCCTTATTTTAATCCTCATTACAAGGGAGTAGGTAATAC
TAAAATGTGTCTTTTTTATGTTTTGGATGTGCTTTGTAATTTTTTTCATTGGAGAATTTATT**ATG**AAAAAATTATCTGTCACATCAAAGAGACA
ATATAATTTATATGCTTCGCCTATTTCTCGACGTTTATCTTTGTTAATGAAGCTCTCATTGGAAACTGTAACAGTTATGTTCTTATTGGGTGCA
TCTCCTGTATTGGCTTCGAATCTTGCGGGTCAGCTTTATTCAATGAGCAATATGCTTGCGACCTATTTGGGTGGTAACGCTAAATATGAGAATG
GTGAATGGACCGCACCTACCTTTAAGGTTAAAACAGTTAACGGTGAAGGCAAGGAAGAAGAGCAAACTTATCAGAATGTAGCGGAAGCTTTGAC
TGGAGTTGGTACGTCTTTCACCAATATAAAAAGTGAGATTGCCAAACAGATTAATCATCTCCAGTCTGATGATTCAGCGGTTATTCATTATGAT
AAGAATAAAGATGAAACTGGCACCATTAATTATGCGAGTGTAACTTTGGGTAAAGGTGAAGATTCTGCAGCTGTTGCCCTTCATAATGTCGCTG
CAGGTAATATTGCTAAGGATTCACGTGATGCAATCAATGGTTCTCAGCTTTATTCTTTGAACGAGCAGTTATTGACCTATTTTGGCGGTGATGC
TGGCTATAAAGATGGGCAATGGATAGCTCCCAAGTTCCATGTTTTGCAGTTCAAGAGTGATGGTAGTTCTGGTGAGAAGGAGAGCTATGATAAT
GTAGCGGCTGCGTTTGAAGGAGTTAACAAAAGTCTTGCAGGTATGAACGAGCGTATTAATAATGTTACTGCTGGCCAGAATGTTTCGTCGAGCA
GTTTAAATTGGAATGAGACGGAGGGAGGTTATGACGCTCGTCATAATGGTGTGGACAGTAAGCTTACGCATGTAGAGAATGGTGACGTATCCGA
AAAATCGAAAGAAGCCGTTAATGGAAGTCAACTATGGAATACGAATGAGAAAGTTGAAGCGGTTGAGAAGGATGTAAAGAATATTGAGAAGAAG
GTACAAGATATTGCTACAGTAGCAGATAGTGCTGTTAAGTATGAGAAAGATAGTACTGGCAAGAAAACGAATGTAATCAAATTAGTTGGTGGGA
GTGAAAGTGAGCCAGTATTGATAGACAATGTAGCGGATGGTAAAATTGAAGCAGACTCTAAGCAGGCAGTCAATGGAGGTCAGTTGCGTGATTA
TACTGAGAAACAGATGAAGATAGTGCTTGATGATGCGAAGAAATATACGGATGAACGCTTCAATGATGTCGTCAATAATGGTATTAATGAGGCT
AAAGCTTATACAGATGTGAAGTTTGAGGCTTTAAGTTACACTGTTGAGGAAGTCCGGAAAGAAGCAAGACAAGCAGCGGCTATTGGTTTAGCAG
TATCTAACTTACGTTACTATGATATACCAGGATCTTTAAGTCTTTCATTTGGTACGGGTATATGGCGTAGTCAGTCTGCATTTGCTATTGGTGC
TGGTTATACATCTGAAGATGGCAATATTCGTTCTAATTTATCTATCACGAGTTCTGGTGGTCAGTGGGGAGTAGGCGCAGGGATTACTTTGAGA
CTGAAA**TGA**TAAAAAAACTAATATTATGATAGAAAAACGAAGTATTTTGATAAATATTCTGTTCTTCCTTGCCTTATTAGGCAAGGGAGAAAGT
TTTGCTGATGAAAACGATAGTGTTTATACGGTGCATCCACCGCATTTATCTATTCCTAATGGGGTAGCGGGTGAAACACGTCGAATCATCATGC
AGTTTTATTATTGGACTTTAATTTGTGATGAAAAACAAAAGCTTAGGCAAGGCATATGTAATGTGACGCAAACTGTCCATGATAAGGAAGGCAA
TACTATTTTCAGTTGGTCTCTTGTTTCTACGAAAAA

protein sequence

MKKLSVTSKRQYNLYASPISRRLSLLMKLSLETVTVMFLLGASPVLASNLAGQLYSMSNMLATYLGGNAKYENGEWTAPTFKVKTVNGEGKEEE
QTYQNVAEALTGVGTSFTNIKSEIAKQINHLQSDDSAVIHYDKNKDETGTINYASVTLGKGEDSAAVALHNVAAGNIAKDSRDAINGSQLYSLN
EQLLTYFGGDAGYKDGQWIAPKFHVLQFKSDGSSGEKESYDNVAAAFEGVNKSLAGMNERINNVTAGQNVSSSSLNWNETEGGYDARHNGVDSK
LTHVENGDVSEKSKEAVNGSQLWNTNEKVEAVEKDVKNIEKKVQDIATVADSAVKYEKDSTGKKTNVIKLVGGSESEPVLIDNVADGKIEADSK
QAVNGGQLRDYTEKQMKIVLDDAKKYTDERFNDVVNNGINEAKAYTDVKFEALSYTVEEVRKEARQAAAIGLAVSNLRYYDIPGSLSLSFGTGI
WRSQSAFAIGAGYTSEDGNIRSNLSITSSGGQWGVGAGITLRLK-

(D) BadA S29

gene sequence

CTGAATTTAGAGAGTGTAAGCTTTTATAGAAGCGTGCTGTTCTCTTTGAAAAGGAATGGTATTGTTCACAAAAAGTACTGTTTTTATTATGAAC
TAAAAAAATTTATTTTTAGCTTGCTATTTTACTCAATAGAGGATAGTGATACAGAAGGTATATCAGTATACTCATTTTAATTATAACTTCAAAA
GGGGAGGAAGTAATGCGTAAAAGACGAAACGCCACTCTAAAAGCAAATTTACATACCGCATCACACTCAATATAAAGAAACACTCGTAACAGAA
ATCAACTAAGCATACAGATTTCTTTTAAATATTCTTCAAATTCTCTTATTATTAAGAAAAGATGCTCCTTAATGAAAAAATTTTTTAATAAAAC
AGATAGCAATAAAAGAATGATTGAAATATTATTTAAACAACACCACCCTAACGTAAAACGTCTTAATATTTAAAACAGAAAAATTCTTTTTTAA
GTACACAACAAAAACAACCGCTCAACCCCTATTACAATCCAAATGCGCTATTTACACGCTTCCTACCAAGCTTTCGCATTCAGATTTCATTACA
GAAAGTACACACAAAATAAAAATAAAGACTCAAAACGTTCCCAATTTGACCACCCTCCTTATTTTAATCCTCATTACAAGGGAGTAGGTAATAC
TAAAATGTGTCTTTTTTATGTTTTGGATGTGCTTTGTAATTTTTTTCATTGGAGAATTTATT**ATG**AAAAAATTATCTGTCACATCAAAGAGACA
ATATAATTTATATGCTTCGCCTATTTCTCGACGTTTATCTTTGTTAATGAAGCTCTCATTGGAAACTGTAACAGTTATGTTCTTATTGGGTGCA
TCTCCTGTATTGGCTTCGAATCTTGCGTCTCAGCTTTATTCTTTGAACGAGCAGTTATTGACCTATTTTGGCGGTGATGCTGGCTATAAAGATG
GGCAATGGATAGCTCCCAAGTTCCATGTTTTGCAGTTCAAGAGTGATGGTAGTTCTGGTGAGAAGGAGAGCTATGATAATGTAGCGGCTGCGTT
TGAAGGAGTTAACAAAAGTCTTGCAGGTATGAACGAGCGTATTAATAATGTTACTGCTGGCCAGAATGTTTCGTCGAGCAGTTTAAATTGGAAT
GAGACGGAGGGAGGTTATGACGCTCGTCATAATGGTGTGGACAGTAAGCTTACGCATGTAGAGAATGGTGACGTATCCGAAAAATCGAAAGAAG
CCGTTAATGGAAGTCAACTATGGAATACGAATGAGAAAGTTGAAGCGGTTGAGAAGGATGTAAAGAATATTGAGAAGAAGGTACAAGATATTGC
TACAGTAGCAGATAGTGCTGTTAAGTATGAGAAAGATAGTACTGGCAAGAAAACGAATGTAATCAAATTAGTTGGTGGGAGTGAAAGTGAGCCA
GTATTGATAGACAATGTAGCGGATGGTAAAATTGAAGCAGACTCTAAGCAGGCAGTCAATGGAGGTCAGTTGCGTGATTATACTGAGAAACAGA
TGAAGATAGTGCTTGATGATGCGAAGAAATATACGGATGAACGCTTCAATGATGTCGTCAATAATGGTATTAATGAGGCTAAAGCTTATACAGA
TGTGAAGTTTGAGGCTTTAAGTTACACTGTTGAGGAAGTCCGGAAAGAAGCAAGACAAGCAGCGGCTATTGGTTTAGCAGTATCTAACTTACGT
TACTATGATATACCAGGATCTTTAAGTCTTTCATTTGGTACGGGTATATGGCGTAGTCAGTCTGCATTTGCTATTGGTGCTGGTTATACATCTG
AAGATGGCAATATTCGTTCTAATTTATCTATCACGAGTTCTGGTGGTCAGTGGGGAGTAGGCGCAGGGATTACTTTGAGACTGAAA**TGA**TAAAA
AAACTAATATTATGATAGAAAAACGAAGTATTTTGATAAATATTCTGTTCTTCCTTGCCTTATTAGGCAAGGGAGAAAGTTTTGCTGATGAAAA
CGATAGTGTTTATACGGTGCATCCACCGCATTTATCTATTCCTAATGGGGTAGCGGGTGAAACACGTCGAATCATCATGCAGTTTTATTATTGG
ACTTTAATTTGTGATGAAAAACAAAAGCTTAGGCAAGGCATATGTAATGTGACGCAAACTGTCCATGATAAGGAAGGCAATACTATTTTCAGTT
GGTCTCTTGTTTCTACGAAAAA

protein sequence

MKKLSVTSKRQYNLYASPISRRLSLLMKLSLETVTVMFLLGASPVLASNLASQLYSLNEQLLTYFGGDAGYKDGQWIAPKFHVLQFKSDGSSGE
KESYDNVAAAFEGVNKSLAGMNERINNVTAGQNVSSSSLNWNETEGGYDARHNGVDSKLTHVENGDVSEKSKEAVNGSQLWNTNEKVEAVEKDV
KNIEKKVQDIATVADSAVKYEKDSTGKKTNVIKLVGGSESEPVLIDNVADGKIEADSKQAVNGGQLRDYTEKQMKIVLDDAKKYTDERFNDVVN
NGINEAKAYTDVKFEALSYTVEEVRKEARQAAAIGLAVSNLRYYDIPGSLSLSFGTGIWRSQSAFAIGAGYTSEDGNIRSNLSITSSGGQWGVG
AGITLRLK-

(E) BadA HNS30

gene sequence

CTGAATTTAGAGAGTGTAAGCTTTTATAGAAGCGTGCTGTTCTCTTTGAAAAGGAATGGTATTGTTCACAAAAAGTACTGTTTTTATTATGAAC
TAAAAAAATTTATTTTTAGCTTGCTATTTTACTCAATAGAGGATAGTGATACAGAAGGTATATCAGTATACTCATTTTAATTATAACTTCAAAA
GGGGAGGAAGTAATGCGTAAAAGACGAAACGCCACTCTAAAAGCAAATTTACATACCGCATCACACTCAATATAAAGAAACACTCGTAACAGAA
ATCAACTAAGCATACAGATTTCTTTTAAATATTCTTCAAATTCTCTTATTATTAAGAAAAGATGCTCCTTAATGAAAAAATTTTTTAATAAAAC
AGATAGCAATAAAAGAATGATTGAAATATTATTTAAACAACACCACCCTAACGTAAAACGTCTTAATATTTAAAACAGAAAAATTCTTTTTTAA
GTACACAACAAAAACAACCGCTCAACCCCTATTACAATCCAAATGCGCTATTTACACGCTTCCTACCAAGCTTTCGCATTCAGATTTCATTACA
GAAAGTACACACAAAATAAAAATAAAGACTCAAAACGTTCCCAATTTGACCACCCTCCTTATTTTAATCCTCATTACAAGGGAGTAGGTAATAC
TAAAATGTGTCTTTTTTATGTTTTGGATGTGCTTTGTAATTTTTTTCATTGGAGAATTTATT**ATG**AAAAAATTATCTGTCACATCAAAGAGACA
ATATAATTTATATGCTTCGCCTATTTCTCGACGTTTATCTTTGTTAATGAAGCTCTCATTGGAAACTGTAACAGTTATGTTCTTATTGGGTGCA
TCTCCTGTATTGGCTTCGAATCTTGCGCTTACAGGAGCAAAGAATCTGAGTCAAAACTCTCCAGGTGTAAATTACTCTAAAGGTAGCCATGGTA
GTATTGTTCTCTCTGGTGATGATGATTTTTGCGGTGCGGATTATGTTCTTGGTCGTGGAGGCAATTCTACTGTACGTAATGGGATTCCAATAAG
TGTAGAAGAAGAATATGAGAGATTTGTCAAACAAAAATTAATGAATAATGCTACTTCTCCTTATAGTCAGAGTTCAGAGCAACAAGTTTGGACT
GGTGATGGGCTAACAAGCAAAGGTTCGGGTTATATGGGAGGGAAGTCGACTGACGGTGATAAAAATATCTTGCCTGAGGCTTATGGTATATATT
CTTTTGCAACTGGTTGTGGTTCTTCTGCGCAGGGGAATTATTCAGTTGCATTTGGTGCAAATGCAACTGCACTTACTGGGGGGTCGCAAGCTTT
TGGTGTTGCTGCACTTGCAAGTGGAAGGGTAAGTGTTGCTATTGGTGTAGGGTCAGAAGCGACGGGAGAGGCTGGAGTTTCTTTGGGTGGACTC
TCAAAGGCAGCTGGTGCTCGTAGTGTTGCTATAGGGACGCGGGCTAAAGCTCAGGGTGAAGAATCTATTGCGATAGGTAGTAGCGTAAAGAATG
GTGATAAGGACGGTTCAGCTGTAGCGCAGGGTGCAAAAGCGATTGCTATAGGTTCTAATTCTATTAGTTTTCAGCACTATGCAGTTGCGGTTGG
TGCTAAAGCCCATGCTCTTCTCTCGAAAACTGTTGCCTTGGGTTATGATTCTGTTGCTGATGTTGATGCTGGCATTAGAGGTTATGATCCTGTG
GAGGATGAGCCATCGAAAGACGTTAGTTTTGTATGGAAAAGCTCTCTAGGTGCTGTTAGTGTTGGTAATCGTAAAGAAGGCTTAACGCGACAAA
TTATAGGAGTTGCAGCTGGTACTGAAGACACTGATGCAGTAAATGTTGCACAGCTAAAAGCATTAAGGGGAATGATATCAGAAAAAGGAGGTTG
GAATCTTACTGTTAATAATGACAATAATACAGTTGTTAGCTCAGGTGGTGCATTAGATTTGTCATCTGGAAGTAAAAATCTCAAAATTGTAAAA
GATGGAAAAAAGAATAATGTAACCTTTGATGTCGCTAGGGATCTCACGTTAAAGAGCATAAAATTAGACGGTGTTACTTTAAATGAAACAGGTT
TATTTATTGCAAACGGACCACAAATCACCGCTTCAGGTATTAATGCTGGTAGTCAAAAACTTACGCATGTAGAGAATGGTGACGTATCCGAAAA
ATCGAAAGAAGCCGTTAATGGAAGTCAACTATGGAATACGAATGAGAAAGTTGAAGCGGTTGAGAAGGATGTAAAGAATATTGAGAAGAAGGTA
CAAGATATTGCTACAGTAGCAGATAGTGCTGTTAAGTATGAGAAAGATAGTACTGGCAAGAAAACGAATGTAATCAAATTAGTTGGTGGGAGTG
AAAGTGAGCCAGTATTGATAGACAATGTAGCGGATGGTAAAATTGAAGCAGACTCTAAGCAGGCAGTCAATGGAGGTCAGTTGCGTGATTATAC
TGAGAAACAGATGAAGATAGTGCTTGATGATGCGAAGAAATATACGGATGAACGCTTCAATGATGTCGTCAATAATGGTATTAATGAGGCTAAA
GCTTATACAGATGTGAAGTTTGAGGCTTTAAGTTACACTGTTGAGGAAGTCCGGAAAGAAGCAAGACAAGCAGCGGCTATTGGTTTAGCAGTAT
CTAACTTACGTTACTATGATATACCAGGATCTTTAAGTCTTTCATTTGGTACGGGTATATGGCGTAGTCAGTCTGCATTTGCTATTGGTGCTGG
TTATACATCTGAAGATGGCAATATTCGTTCTAATTTATCTATCACGAGTTCTGGTGGTCAGTGGGGAGTAGGCGCAGGGATTACTTTGAGACTG
AAA**TGA**TAAAAAAACTAATATTATGATAGAAAAACGAAGTATTTTGATAAATATTCTGTTCTTCCTTGCCTTATTAGGCAAGGGAGAAAGTTTT
GCTGATGAAAACGATAGTGTTTATACGGTGCATCCACCGCATTTATCTATTCCTAATGGGGTAGCGGGTGAAACACGTCGAATCATCATGCAGT
TTTATTATTGGACTTTAATTTGTGATGAAAAACAAAAGCTTAGGCAAGGCATATGTAATGTGACGCAAACTGTCCATGATAAGGAAGGCAATAC
TATTTTCAGTTGGTCTCTTGTTTCTACGAAAAA

protein sequence

MKKLSVTSKRQYNLYASPISRRLSLLMKLSLETVTVMFLLGASPVLASNLALTGAKNLSQNSPGVNYSKGSHGSIVLSGDDDFCGADYVLGRGG
NSTVRNGIPISVEEEYERFVKQKLMNNATSPYSQSSEQQVWTGDGLTSKGSGYMGGKSTDGDKNILPEAYGIYSFATGCGSSAQGNYSVAFGAN
ATALTGGSQAFGVAALASGRVSVAIGVGSEATGEAGVSLGGLSKAAGARSVAIGTRAKAQGEESIAIGSSVKNGDKDGSAVAQGAKAIAIGSNS
ISFQHYAVAVGAKAHALLSKTVALGYDSVADVDAGIRGYDPVEDEPSKDVSFVWKSSLGAVSVGNRKEGLTRQIIGVAAGTEDTDAVNVAQLKA
LRGMISEKGGWNLTVNNDNNTVVSSGGALDLSSGSKNLKIVKDGKKNNVTFDVARDLTLKSIKLDGVTLNETGLFIANGPQITASGINAGSQKL
THVENGDVSEKSKEAVNGSQLWNTNEKVEAVEKDVKNIEKKVQDIATVADSAVKYEKDSTGKKTNVIKLVGGSESEPVLIDNVADGKIEADSKQ
AVNGGQLRDYTEKQMKIVLDDAKKYTDERFNDVVNNGINEAKAYTDVKFEALSYTVEEVRKEARQAAAIGLAVSNLRYYDIPGSLSLSFGTGIW
RSQSAFAIGAGYTSEDGNIRSNLSITSSGGQWGVGAGITLRLK-

(F) BadA S30

gene sequence

CTGAATTTAGAGAGTGTAAGCTTTTATAGAAGCGTGCTGTTCTCTTTGAAAAGGAATGGTATTGTTCACAAAAAGTACTGTTTTTATTATGAAC
TAAAAAAATTTATTTTTAGCTTGCTATTTTACTCAATAGAGGATAGTGATACAGAAGGTATATCAGTATACTCATTTTAATTATAACTTCAAAA
GGGGAGGAAGTAATGCGTAAAAGACGAAACGCCACTCTAAAAGCAAATTTACATACCGCATCACACTCAATATAAAGAAACACTCGTAACAGAA
ATCAACTAAGCATACAGATTTCTTTTAAATATTCTTCAAATTCTCTTATTATTAAGAAAAGATGCTCCTTAATGAAAAAATTTTTTAATAAAAC
AGATAGCAATAAAAGAATGATTGAAATATTATTTAAACAACACCACCCTAACGTAAAACGTCTTAATATTTAAAACAGAAAAATTCTTTTTTAA
GTACACAACAAAAACAACCGCTCAACCCCTATTACAATCCAAATGCGCTATTTACACGCTTCCTACCAAGCTTTCGCATTCAGATTTCATTACA
GAAAGTACACACAAAATAAAAATAAAGACTCAAAACGTTCCCAATTTGACCACCCTCCTTATTTTAATCCTCATTACAAGGGAGTAGGTAATAC
TAAAATGTGTCTTTTTTATGTTTTGGATGTGCTTTGTAATTTTTTTCATTGGAGAATTTATT**ATG**AAAAAATTATCTGTCACATCAAAGAGACA
ATATAATTTATATGCTTCGCCTATTTCTCGACGTTTATCTTTGTTAATGAAGCTCTCATTGGAAACTGTAACAGTTATGTTCTTATTGGGTGCA
TCTCCTGTATTGGCTTCGAATCTTGCGCTTACGCATGTAGAGAATGGTGACGTATCCGAAAAATCGAAAGAAGCCGTTAATGGAAGTCAACTAT
GGAATACGAATGAGAAAGTTGAAGCGGTTGAGAAGGATGTAAAGAATATTGAGAAGAAGGTACAAGATATTGCTACAGTAGCAGATAGTGCTGT
TAAGTATGAGAAAGATAGTACTGGCAAGAAAACGAATGTAATCAAATTAGTTGGTGGGAGTGAAAGTGAGCCAGTATTGATAGACAATGTAGCG
GATGGTAAAATTGAAGCAGACTCTAAGCAGGCAGTCAATGGAGGTCAGTTGCGTGATTATACTGAGAAACAGATGAAGATAGTGCTTGATGATG
CGAAGAAATATACGGATGAACGCTTCAATGATGTCGTCAATAATGGTATTAATGAGGCTAAAGCTTATACAGATGTGAAGTTTGAGGCTTTAAG
TTACACTGTTGAGGAAGTCCGGAAAGAAGCAAGACAAGCAGCGGCTATTGGTTTAGCAGTATCTAACTTACGTTACTATGATATACCAGGATCT
TTAAGTCTTTCATTTGGTACGGGTATATGGCGTAGTCAGTCTGCATTTGCTATTGGTGCTGGTTATACATCTGAAGATGGCAATATTCGTTCTA
ATTTATCTATCACGAGTTCTGGTGGTCAGTGGGGAGTAGGCGCAGGGATTACTTTGAGACTGAAA**TGA**TAAAAAAACTAATATTATGATAGAAA
AACGAAGTATTTTGATAAATATTCTGTTCTTCCTTGCCTTATTAGGCAAGGGAGAAAGTTTTGCTGATGAAAACGATAGTGTTTATACGGTGCA
TCCACCGCATTTATCTATTCCTAATGGGGTAGCGGGTGAAACACGTCGAATCATCATGCAGTTTTATTATTGGACTTTAATTTGTGATGAAAAA
CAAAAGCTTAGGCAAGGCATATGTAATGTGACGCAAACTGTCCATGATAAGGAAGGCAATACTATTTTCAGTTGGTCTCTTGTTTCTACGAAAA
A

protein sequence

MKKLSVTSKRQYNLYASPISRRLSLLMKLSLETVTVMFLLGASPVLASNLALTHVENGDVSEKSKEAVNGSQLWNTNEKVEAVEKDVKNIEKKV
QDIATVADSAVKYEKDSTGKKTNVIKLVGGSESEPVLIDNVADGKIEADSKQAVNGGQLRDYTEKQMKIVLDDAKKYTDERFNDVVNNGINEAK
AYTDVKFEALSYTVEEVRKEARQAAAIGLAVSNLRYYDIPGSLSLSFGTGIWRSQSAFAIGAGYTSEDGNIRSNLSITSSGGQWGVGAGITLRL
K-

(G) BadA D16S28

gene sequence

GAATTCCTGAATTTAGAGAGTGTAAGCTTTTATAGAAGCGTGCTGTTCTCTTTGAAAAGGAATGGTATTGTTCACAAAAAGTACTGTTTTTATT
ATGAACTAAAAAAATTTATTTTTAGCTTGCTATTTTACTCAATAGAGGATAGTGATACAGAAGGTATATCAGTATACTCATTTTAATTATAACT
TCAAAAGGGGAGGAAGTAATGCGTAAAAGACGAAACGCCACTCTAAAAGCAAATTTACATACCGCATCACACTCAATATAAAGAAACACTCGTA
ACAGAAATCAACTAAGCATACAGATTTCTTTTAAATATTCTTCAAATTCTCTTATTATTAAGAAAAGATGCTCCTTAATGAAAAAATTTTTTAA
TAAAACAGATAGCAATAAAAGAATGATTGAAATATTATTTAAACAACACCACCCTAACGTAAAACGTCTTAATATTTAAAACAGAAAAATTCTT
TTTTAAGTACACAACAAAAACAACCGCTCAACCCCTATTACAATCCAAATGCGCTATTTACACGCTTCCTACCAAGCTTTCGCATTCAGATTTC
ATTACAGAAAGTACACACAAAATAAAAATAAAGACTCAAAACGTTCCCAATTTGACCACCCTCCTTATTTTAATCCTCATTACAAGGGAGTAGG
TAATACTAAAATGTGTCTTTTTTATGTTTTGGATGTGCTTTGTAATTTTTTTCATTGGAGAATTTATT**ATG**AAAAAATTATCTGTCACATCAAA
GAGACAATATAATTTATATGCTTCGCCTATTTCTCGACGTTTATCTTTGTTAATGAAGCTCTCATTGGAAACTGTAACAGTTATGTTCTTATTG
GGTGCATCTCCTGTATTGGCTTCGAATCTTGCGGGTCAGCTTTATTCTCTGAACGAGCAACTTGCGACCTATTTTGGCGGCGGTGCTAAGTACG
AGAATGGCCAATGGACTGCACCTACCTTTAAGGTTAAAACAGTTAACGGTGAAGGCAAGGAAGAAGAGCAGACTTATCAGAATGTAGCAGCAGC
TTTTGAAGGAGTTGGTACGTCTTTCACCAATATAAAAAGTGAGATTACTAAACAGATTAATAATGAGATTATCAATGTAAAAGGTGATAGTCTT
GTTAAGAGAGATCTCGCTACGAATCTCATCACCATTGGTAAAGAAATAGAAGGCAGTGTAATCAATATTGCTAATAAGAGTGGTGAAGCTCGGA
CCATTTCTGGTGTTAAGGAAGCAGTAAAAGATAATGAAGCTGTTAACAAAGGGCAGCTTTATTCAATGAGCAATATGCTTGCGACCTATTTGGG
TGGTAACGCTAAATATGAGAATGGTGAATGGACCGCACCTACCTTTAAGGTTAAAACAGTTAACGGTGAAGGCAAGGAAGAAGAGCAAACTTAT
CAGAATGTAGCGGAAGCTTTGACTGGAGTTGGTACGTCTTTCACCAATATAAAAAGTGAGATTGCCAAACAGATTAATCATCTCCAGTCTGATG
ATTCAGCGGTTATTCATTATGATAAGAATAAAGATGAAACTGGCACCATTAATTATGCGAGTGTAACTTTGGGTAAAGGTGAAGATTCTGCAGC
TGTTGCCCTTCATAATGTCGCTGCAGGTAATATTGCTAAGGATTCACGTGATGCAATCAATGGTTCTCAGCTTTATTCTTTGAACGAGCAGTTA
TTGACCTATTTTGGCGGTGATGCTGGCTATAAAGATGGGCAATGGATAGCTCCCAAGTTCCATGTTTTGCAGTTCAAGAGTGATGGTAGTTCTG
GTGAGAAGGAGAGCTATGATAATGTAGCGGCTGCGTTTGAAGGAGTTAACAAAAGTCTTGCAGGTATGAACGAGCGTATTAATAATGTTACTGC
TGGCCAGAATGTTTCGTCGAGCAGTTTAAATTGGAATGAGACGGAGGGAGGTTATGACGCTCGTCATAATGGTGTGGACAGTAAGCTTACGCAT
GTAGAGAATGGTGACGTATCCGAAAAATCGAAAGAAGCCGTTAATGGAAGTCAACTATGGAATACGAATGAGAAAGTTGAAGCGGTTGAGAAGG
ATGTAAAGAATATTGAGAAGAAGGTACAAGATATTGCTACAGTAGCAGATAGTGCTGTTAAGTATGAGAAAGATAGTACTGGCAAGAAAACGAA
TGTAATCAAATTAGTTGGTGGGAGTGAAAGTGAGCCAGTATTGATAGACAATGTAGCGGATGGTAAAATTGAAGCAGACTCTAAGCAGGCAGTC
AATGGAGGTCAGTTGCGTGATTATACTGAGAAACAGATGAAGATAGTGCTTGATGATGCGAAGAAATATACGGATGAACGCTTCAATGATGTCG
TCAATAATGGTATTAATGAGGCTAAAGCTTATACAGATGTGAAGTTTGAGGCTTTAAGTTACACTGTTGAGGAAGTCCGGAAAGAAGCAAGACA
AGCAGCGGCTATTGGTTTAGCAGTATCTAACTTACGTTACTATGATATACCAGGATCTTTAAGTCTTTCATTTGGTACGGGTATATGGCGTAGT
CAGTCTGCATTTGCTATTGGTGCTGGTTATACATCTGAAGATGGCAATATTCGTTCTAATTTATCTATCACGAGTTCTGGTGGTCAGTGGGGAG
TAGGCGCAGGGATTACTTTGAGACTGAAA**TGA**TAAAAAAACTAATATTATGATAGAAAAACGAAGTATTTTGATAAATATTCTGTTCTTCCTTG
CCTTATTAGGCAAGGGAGAAAGTTTTGCTGATGAAAACGATAGTGTTTATACGGTGCATCCACCGCATTTATCTATTCCTAATGGGGTAGCGGG
TGAAACACGTCGAATCATCATGCAGTTTTATTATTGGACTTTAATTTGTGATGAAAAACAAAAGCTTAGGCAAGGCATATGTAATGTGACGCAA
ACTGTCCATGATAAGGAAGGCAATACTATTTTCAGTTGGTCTCTTGTTTCTACGAAAAAGAATTC

protein sequence

MKKLSVTSKRQYNLYASPISRRLSLLMKLSLETVTVMFLLGASPVLASNLAGQLYSLNEQLATYFGGGAKYENGQWTAPTFKVKTVNGEGKEEE
QTYQNVAAAFEGVGTSFTNIKSEITKQINNEIINVKGDSLVKRDLATNLITIGKEIEGSVINIANKSGEARTISGVKEAVKDNEAVNKGQLYSM
SNMLATYLGGNAKYENGEWTAPTFKVKTVNGEGKEEEQTYQNVAEALTGVGTSFTNIKSEIAKQINHLQSDDSAVIHYDKNKDETGTINYASVT
LGKGEDSAAVALHNVAAGNIAKDSRDAINGSQLYSLNEQLLTYFGGDAGYKDGQWIAPKFHVLQFKSDGSSGEKESYDNVAAAFEGVNKSLAGM
NERINNVTAGQNVSSSSLNWNETEGGYDARHNGVDSKLTHVENGDVSEKSKEAVNGSQLWNTNEKVEAVEKDVKNIEKKVQDIATVADSAVKYE
KDSTGKKTNVIKLVGGSESEPVLIDNVADGKIEADSKQAVNGGQLRDYTEKQMKIVLDDAKKYTDERFNDVVNNGINEAKAYTDVKFEALSYTV
EEVRKEARQAAAIGLAVSNLRYYDIPGSLSLSFGTGIWRSQSAFAIGAGYTSEDGNIRSNLSITSSGGQWGVGAGITLRLK-

(H) BadA D19S28

gene sequence

GAATTCCTGAATTTAGAGAGTGTAAGCTTTTATAGAAGCGTGCTGTTCTCTTTGAAAAGGAATGGTATTGTTCACAAAAAGTACTGTTTTTATT
ATGAACTAAAAAAATTTATTTTTAGCTTGCTATTTTACTCAATAGAGGATAGTGATACAGAAGGTATATCAGTATACTCATTTTAATTATAACT
TCAAAAGGGGAGGAAGTAATGCGTAAAAGACGAAACGCCACTCTAAAAGCAAATTTACATACCGCATCACACTCAATATAAAGAAACACTCGTA
ACAGAAATCAACTAAGCATACAGATTTCTTTTAAATATTCTTCAAATTCTCTTATTATTAAGAAAAGATGCTCCTTAATGAAAAAATTTTTTAA
TAAAACAGATAGCAATAAAAGAATGATTGAAATATTATTTAAACAACACCACCCTAACGTAAAACGTCTTAATATTTAAAACAGAAAAATTCTT
TTTTAAGTACACAACAAAAACAACCGCTCAACCCCTATTACAATCCAAATGCGCTATTTACACGCTTCCTACCAAGCTTTCGCATTCAGATTTC
ATTACAGAAAGTACACACAAAATAAAAATAAAGACTCAAAACGTTCCCAATTTGACCACCCTCCTTATTTTAATCCTCATTACAAGGGAGTAGG
TAATACTAAAATGTGTCTTTTTTATGTTTTGGATGTGCTTTGTAATTTTTTTCATTGGAGAATTTATT**ATG**AAAAAATTATCTGTCACATCAAA
GAGACAATATAATTTATATGCTTCGCCTATTTCTCGACGTTTATCTTTGTTAATGAAGCTCTCATTGGAAACTGTAACAGTTATGTTCTTATTG
GGTGCATCTCCTGTATTGGCTTCGAATCTTGCGGGCCAGCTTGATGCCAATATCAGTAAAGTTAATAATAATGTAACGAATAAGTTTAATGAAC
TTACTCAAAGCATAACGAATGTTACGCAACAGGTAAAAGGCGATGCCTTATTATGGAGCGATGAAGCCAATGCTTTTGTGGCGCGTCATGAAAA
GAGTAAGTTAGAAAAAGGCGTATCTAAAGCGACACAAGAAAATAGCAAGATTACGTATCTGTTAGATGGTGATATTTCGAAAGGTTCCACGGAT
GCCGTTACCGGTGGTCAGCTTTATTCAATGAGCAATATGCTTGCGACCTATTTGGGTGGTAACGCTAAATATGAGAATGGTGAATGGACCGCAC
CTACCTTTAAGGTTAAAACAGTTAACGGTGAAGGCAAGGAAGAAGAGCAAACTTATCAGAATGTAGCGGAAGCTTTGACTGGAGTTGGTACGTC
TTTCACCAATATAAAAAGTGAGATTGCCAAACAGATTAATCATCTCCAGTCTGATGATTCAGCGGTTATTCATTATGATAAGAATAAAGATGAA
ACTGGCACCATTAATTATGCGAGTGTAACTTTGGGTAAAGGTGAAGATTCTGCAGCTGTTGCCCTTCATAATGTCGCTGCAGGTAATATTGCTA
AGGATTCACGTGATGCAATCAATGGTTCTCAGCTTTATTCTTTGAACGAGCAGTTATTGACCTATTTTGGCGGTGATGCTGGCTATAAAGATGG
GCAATGGATAGCTCCCAAGTTCCATGTTTTGCAGTTCAAGAGTGATGGTAGTTCTGGTGAGAAGGAGAGCTATGATAATGTAGCGGCTGCGTTT
GAAGGAGTTAACAAAAGTCTTGCAGGTATGAACGAGCGTATTAATAATGTTACTGCTGGCCAGAATGTTTCGTCGAGCAGTTTAAATTGGAATG
AGACGGAGGGAGGTTATGACGCTCGTCATAATGGTGTGGACAGTAAGCTTACGCATGTAGAGAATGGTGACGTATCCGAAAAATCGAAAGAAGC
CGTTAATGGAAGTCAACTATGGAATACGAATGAGAAAGTTGAAGCGGTTGAGAAGGATGTAAAGAATATTGAGAAGAAGGTACAAGATATTGCT
ACAGTAGCAGATAGTGCTGTTAAGTATGAGAAAGATAGTACTGGCAAGAAAACGAATGTAATCAAATTAGTTGGTGGGAGTGAAAGTGAGCCAG
TATTGATAGACAATGTAGCGGATGGTAAAATTGAAGCAGACTCTAAGCAGGCAGTCAATGGAGGTCAGTTGCGTGATTATACTGAGAAACAGAT
GAAGATAGTGCTTGATGATGCGAAGAAATATACGGATGAACGCTTCAATGATGTCGTCAATAATGGTATTAATGAGGCTAAAGCTTATACAGAT
GTGAAGTTTGAGGCTTTAAGTTACACTGTTGAGGAAGTCCGGAAAGAAGCAAGACAAGCAGCGGCTATTGGTTTAGCAGTATCTAACTTACGTT
ACTATGATATACCAGGATCTTTAAGTCTTTCATTTGGTACGGGTATATGGCGTAGTCAGTCTGCATTTGCTATTGGTGCTGGTTATACATCTGA
AGATGGCAATATTCGTTCTAATTTATCTATCACGAGTTCTGGTGGTCAGTGGGGAGTAGGCGCAGGGATTACTTTGAGACTGAAA**TGA**TAAAAA
AACTAATATTATGATAGAAAAACGAAGTATTTTGATAAATATTCTGTTCTTCCTTGCCTTATTAGGCAAGGGAGAAAGTTTTGCTGATGAAAAC
GATAGTGTTTATACGGTGCATCCACCGCATTTATCTATTCCTAATGGGGTAGCGGGTGAAACACGTCGAATCATCATGCAGTTTTATTATTGGA
CTTTAATTTGTGATGAAAAACAAAAGCTTAGGCAAGGCATATGTAATGTGACGCAAACTGTCCATGATAAGGAAGGCAATACTATTTTCAGTTG
GTCTCTTGTTTCTACGAAAAAGAATTC

protein sequence

MKKLSVTSKRQYNLYASPISRRLSLLMKLSLETVTVMFLLGASPVLASNLAGQLDANISKVNNNVTNKFNELTQSITNVTQQVKGDALLWSDEA
NAFVARHEKSKLEKGVSKATQENSKITYLLDGDISKGSTDAVTGGQLYSMSNMLATYLGGNAKYENGEWTAPTFKVKTVNGEGKEEEQTYQNVA
EALTGVGTSFTNIKSEIAKQINHLQSDDSAVIHYDKNKDETGTINYASVTLGKGEDSAAVALHNVAAGNIAKDSRDAINGSQLYSLNEQLLTYF
GGDAGYKDGQWIAPKFHVLQFKSDGSSGEKESYDNVAAAFEGVNKSLAGMNERINNVTAGQNVSSSSLNWNETEGGYDARHNGVDSKLTHVENG
DVSEKSKEAVNGSQLWNTNEKVEAVEKDVKNIEKKVQDIATVADSAVKYEKDSTGKKTNVIKLVGGSESEPVLIDNVADGKIEADSKQAVNGGQ
LRDYTEKQMKIVLDDAKKYTDERFNDVVNNGINEAKAYTDVKFEALSYTVEEVRKEARQAAAIGLAVSNLRYYDIPGSLSLSFGTGIWRSQSAF
AIGAGYTSEDGNIRSNLSITSSGGQWGVGAGITLRLK-

(I) BadA D25S28

gene sequence

GAATTCCTGAATTTAGAGAGTGTAAGCTTTTATAGAAGCGTGCTGTTCTCTTTGAAAAGGAATGGTATTGTTCACAAAAAGTACTGTTTTTATT
ATGAACTAAAAAAATTTATTTTTAGCTTGCTATTTTACTCAATAGAGGATAGTGATACAGAAGGTATATCAGTATACTCATTTTAATTATAACT
TCAAAAGGGGAGGAAGTAATGCGTAAAAGACGAAACGCCACTCTAAAAGCAAATTTACATACCGCATCACACTCAATATAAAGAAACACTCGTA
ACAGAAATCAACTAAGCATACAGATTTCTTTTAAATATTCTTCAAATTCTCTTATTATTAAGAAAAGATGCTCCTTAATGAAAAAATTTTTTAA
TAAAACAGATAGCAATAAAAGAATGATTGAAATATTATTTAAACAACACCACCCTAACGTAAAACGTCTTAATATTTAAAACAGAAAAATTCTT
TTTTAAGTACACAACAAAAACAACCGCTCAACCCCTATTACAATCCAAATGCGCTATTTACACGCTTCCTACCAAGCTTTCGCATTCAGATTTC
ATTACAGAAAGTACACACAAAATAAAAATAAAGACTCAAAACGTTCCCAATTTGACCACCCTCCTTATTTTAATCCTCATTACAAGGGAGTAGG
TAATACTAAAATGTGTCTTTTTTATGTTTTGGATGTGCTTTGTAATTTTTTTCATTGGAGAATTTATT**ATG**AAAAAATTATCTGTCACATCAAA
GAGACAATATAATTTATATGCTTCGCCTATTTCTCGACGTTTATCTTTGTTAATGAAGCTCTCATTGGAAACTGTAACAGTTATGTTCTTATTG
GGTGCATCTCCTGTATTGGCTTCGAATCTTGCGGGGCAGCTTGATACCAATATCAAGAAAGTAGAAGATAAATTAACAGAAGCAGTCGGTAAAG
TTACGCAACAGGTAAAAGGTGATGCTTTATTGTGGAGCAATGAAGATAACGCGTTTGTTGCTGATCATGGTAAAGATAGCGCAAAGACAAAGAG
CAAGATTACACATTTATTAGATGGAAATATTGCGTCTGGCTCAACCGATGCCGTTACCGGTGGTCAACTCTATTCAATGAGCAATATGCTTGCG
ACCTATTTGGGTGGTAACGCTAAATATGAGAATGGTGAATGGACCGCACCTACCTTTAAGGTTAAAACAGTTAACGGTGAAGGCAAGGAAGAAG
AGCAAACTTATCAGAATGTAGCGGAAGCTTTGACTGGAGTTGGTACGTCTTTCACCAATATAAAAAGTGAGATTGCCAAACAGATTAATCATCT
CCAGTCTGATGATTCAGCGGTTATTCATTATGATAAGAATAAAGATGAAACTGGCACCATTAATTATGCGAGTGTAACTTTGGGTAAAGGTGAA
GATTCTGCAGCTGTTGCCCTTCATAATGTCGCTGCAGGTAATATTGCTAAGGATTCACGTGATGCAATCAATGGTTCTCAGCTTTATTCTTTGA
ACGAGCAGTTATTGACCTATTTTGGCGGTGATGCTGGCTATAAAGATGGGCAATGGATAGCTCCCAAGTTCCATGTTTTGCAGTTCAAGAGTGA
TGGTAGTTCTGGTGAGAAGGAGAGCTATGATAATGTAGCGGCTGCGTTTGAAGGAGTTAACAAAAGTCTTGCAGGTATGAACGAGCGTATTAAT
AATGTTACTGCTGGCCAGAATGTTTCGTCGAGCAGTTTAAATTGGAATGAGACGGAGGGAGGTTATGACGCTCGTCATAATGGTGTGGACAGTA
AGCTTACGCATGTAGAGAATGGTGACGTATCCGAAAAATCGAAAGAAGCCGTTAATGGAAGTCAACTATGGAATACGAATGAGAAAGTTGAAGC
GGTTGAGAAGGATGTAAAGAATATTGAGAAGAAGGTACAAGATATTGCTACAGTAGCAGATAGTGCTGTTAAGTATGAGAAAGATAGTACTGGC
AAGAAAACGAATGTAATCAAATTAGTTGGTGGGAGTGAAAGTGAGCCAGTATTGATAGACAATGTAGCGGATGGTAAAATTGAAGCAGACTCTA
AGCAGGCAGTCAATGGAGGTCAGTTGCGTGATTATACTGAGAAACAGATGAAGATAGTGCTTGATGATGCGAAGAAATATACGGATGAACGCTT
CAATGATGTCGTCAATAATGGTATTAATGAGGCTAAAGCTTATACAGATGTGAAGTTTGAGGCTTTAAGTTACACTGTTGAGGAAGTCCGGAAA
GAAGCAAGACAAGCAGCGGCTATTGGTTTAGCAGTATCTAACTTACGTTACTATGATATACCAGGATCTTTAAGTCTTTCATTTGGTACGGGTA
TATGGCGTAGTCAGTCTGCATTTGCTATTGGTGCTGGTTATACATCTGAAGATGGCAATATTCGTTCTAATTTATCTATCACGAGTTCTGGTGG
TCAGTGGGGAGTAGGCGCAGGGATTACTTTGAGACTGAAA**TGA**TAAAAAAACTAATATTATGATAGAAAAACGAAGTATTTTGATAAATATTCT
GTTCTTCCTTGCCTTATTAGGCAAGGGAGAAAGTTTTGCTGATGAAAACGATAGTGTTTATACGGTGCATCCACCGCATTTATCTATTCCTAAT
GGGGTAGCGGGTGAAACACGTCGAATCATCATGCAGTTTTATTATTGGACTTTAATTTGTGATGAAAAACAAAAGCTTAGGCAAGGCATATGTA
ATGTGACGCAAACTGTCCATGATAAGGAAGGCAATACTATTTTCAGTTGGTCTCTTGTTTCTACGAAAAAGAATTC

protein sequence

MKKLSVTSKRQYNLYASPISRRLSLLMKLSLETVTVMFLLGASPVLASNLAGQLDTNIKKVEDKLTEAVGKVTQQVKGDALLWSNEDNAFVADH
GKDSAKTKSKITHLLDGNIASGSTDAVTGGQLYSMSNMLATYLGGNAKYENGEWTAPTFKVKTVNGEGKEEEQTYQNVAEALTGVGTSFTNIKS
EIAKQINHLQSDDSAVIHYDKNKDETGTINYASVTLGKGEDSAAVALHNVAAGNIAKDSRDAINGSQLYSLNEQLLTYFGGDAGYKDGQWIAPK
FHVLQFKSDGSSGEKESYDNVAAAFEGVNKSLAGMNERINNVTAGQNVSSSSLNWNETEGGYDARHNGVDSKLTHVENGDVSEKSKEAVNGSQL
WNTNEKVEAVEKDVKNIEKKVQDIATVADSAVKYEKDSTGKKTNVIKLVGGSESEPVLIDNVADGKIEADSKQAVNGGQLRDYTEKQMKIVLDD
AKKYTDERFNDVVNNGINEAKAYTDVKFEALSYTVEEVRKEARQAAAIGLAVSNLRYYDIPGSLSLSFGTGIWRSQSAFAIGAGYTSEDGNIRS
NLSITSSGGQWGVGAGITLRLK-

(J) BadA D27S29

gene sequence

GAATTCCTGAATTTAGAGAGTGTAAGCTTTTATAGAAGCGTGCTGTTCTCTTTGAAAAGGAATGGTATTGTTCACAAAAAGTACTGTTTTTATT
ATGAACTAAAAAAATTTATTTTTAGCTTGCTATTTTACTCAATAGAGGATAGTGATACAGAAGGTATATCAGTATACTCATTTTAATTATAACT
TCAAAAGGGGAGGAAGTAATGCGTAAAAGACGAAACGCCACTCTAAAAGCAAATTTACATACCGCATCACACTCAATATAAAGAAACACTCGTA
ACAGAAATCAACTAAGCATACAGATTTCTTTTAAATATTCTTCAAATTCTCTTATTATTAAGAAAAGATGCTCCTTAATGAAAAAATTTTTTAA
TAAAACAGATAGCAATAAAAGAATGATTGAAATATTATTTAAACAACACCACCCTAACGTAAAACGTCTTAATATTTAAAACAGAAAAATTCTT
TTTTAAGTACACAACAAAAACAACCGCTCAACCCCTATTACAATCCAAATGCGCTATTTACACGCTTCCTACCAAGCTTTCGCATTCAGATTTC
ATTACAGAAAGTACACACAAAATAAAAATAAAGACTCAAAACGTTCCCAATTTGACCACCCTCCTTATTTTAATCCTCATTACAAGGGAGTAGG
TAATACTAAAATGTGTCTTTTTTATGTTTTGGATGTGCTTTGTAATTTTTTTCATTGGAGAATTTATT**ATG**AAAAAATTATCTGTCACATCAAA
GAGACAATATAATTTATATGCTTCGCCTATTTCTCGACGTTTATCTTTGTTAATGAAGCTCTCATTGGAAACTGTAACAGTTATGTTCTTATTG
GGTGCATCTCCTGTATTGGCTTCGAATCTTGCGGGACAGATTCATACAATCGGTGAGGATGTTGCAAAATTCTTGGGTGGAGATGCAGCTTTTA
AAGATGGCGCTTTTACCGGCCCAACTTATAAGTTGTCGAATATTGATGCAAAGGGTGATGTACAACAGAGTGAGTTTAAAGATATAGGTTCAGC
CTTTGCGGGTCTTGATACGAACATCAAGAATGTCAATAATAATGTAACGAATAAGCTCAGTGAACTTACTCAAAACATAACGACTGTTACGCAA
CAGGTAAAAGGCAATGCCTTATTATGGAGCGATGAAGCTAATGCCTTTGTGGCGCGTCATGAAAAGAGCAAGTTAGAAAAAGGTGCATCTAAAG
CGATACAAGAAAACAGCAAGATTACGTATCTGTTAGATGGTGATGTTTCGAAAGGTTCCACGGATGCCGTTACTGGTGGTCAGCTTTATTCTTT
GAACGAGCAGTTATTGACCTATTTTGGCGGTGATGCTGGCTATAAAGATGGGCAATGGATAGCTCCCAAGTTCCATGTTTTGCAGTTCAAGAGT
GATGGTAGTTCTGGTGAGAAGGAGAGCTATGATAATGTAGCGGCTGCGTTTGAAGGAGTTAACAAAAGTCTTGCAGGTATGAACGAGCGTATTA
ATAATGTTACTGCTGGCCAGAATGTTTCGTCGAGCAGTTTAAATTGGAATGAGACGGAGGGAGGTTATGACGCTCGTCATAATGGTGTGGACAG
TAAGCTTACGCATGTAGAGAATGGTGACGTATCCGAAAAATCGAAAGAAGCCGTTAATGGAAGTCAACTATGGAATACGAATGAGAAAGTTGAA
GCGGTTGAGAAGGATGTAAAGAATATTGAGAAGAAGGTACAAGATATTGCTACAGTAGCAGATAGTGCTGTTAAGTATGAGAAAGATAGTACTG
GCAAGAAAACGAATGTAATCAAATTAGTTGGTGGGAGTGAAAGTGAGCCAGTATTGATAGACAATGTAGCGGATGGTAAAATTGAAGCAGACTC
TAAGCAGGCAGTCAATGGAGGTCAGTTGCGTGATTATACTGAGAAACAGATGAAGATAGTGCTTGATGATGCGAAGAAATATACGGATGAACGC
TTCAATGATGTCGTCAATAATGGTATTAATGAGGCTAAAGCTTATACAGATGTGAAGTTTGAGGCTTTAAGTTACACTGTTGAGGAAGTCCGGA
AAGAAGCAAGACAAGCAGCGGCTATTGGTTTAGCAGTATCTAACTTACGTTACTATGATATACCAGGATCTTTAAGTCTTTCATTTGGTACGGG
TATATGGCGTAGTCAGTCTGCATTTGCTATTGGTGCTGGTTATACATCTGAAGATGGCAATATTCGTTCTAATTTATCTATCACGAGTTCTGGT
GGTCAGTGGGGAGTAGGCGCAGGGATTACTTTGAGACTGAAA**TGA**TAAAAAAACTAATATTATGATAGAAAAACGAAGTATTTTGATAAATATT
CTGTTCTTCCTTGCCTTATTAGGCAAGGGAGAAAGTTTTGCTGATGAAAACGATAGTGTTTATACGGTGCATCCACCGCATTTATCTATTCCTA
ATGGGGTAGCGGGTGAAACACGTCGAATCATCATGCAGTTTTATTATTGGACTTTAATTTGTGATGAAAAACAAAAGCTTAGGCAAGGCATATG
TAATGTGACGCAAACTGTCCATGATAAGGAAGGCAATACTATTTTCAGTTGGTCTCTTGTTTCTACGAAAAAGAATTC

protein sequence

MKKLSVTSKRQYNLYASPISRRLSLLMKLSLETVTVMFLLGASPVLASNLAGQIHTIGEDVAKFLGGDAAFKDGAFTGPTYKLSNIDAKGDVQQ
SEFKDIGSAFAGLDTNIKNVNNNVTNKLSELTQNITTVTQQVKGNALLWSDEANAFVARHEKSKLEKGASKAIQENSKITYLLDGDVSKGSTDA
VTGGQLYSLNEQLLTYFGGDAGYKDGQWIAPKFHVLQFKSDGSSGEKESYDNVAAAFEGVNKSLAGMNERINNVTAGQNVSSSSLNWNETEGGY
DARHNGVDSKLTHVENGDVSEKSKEAVNGSQLWNTNEKVEAVEKDVKNIEKKVQDIATVADSAVKYEKDSTGKKTNVIKLVGGSESEPVLIDNV
ADGKIEADSKQAVNGGQLRDYTEKQMKIVLDDAKKYTDERFNDVVNNGINEAKAYTDVKFEALSYTVEEVRKEARQAAAIGLAVSNLRYYDIPG
SLSLSFGTGIWRSQSAFAIGAGYTSEDGNIRSNLSITSSGGQWGVGAGITLRLK-

Figure S2. Control of bacterial numbers inoculated in Fn binding assays. qPCR results showing the logarithmic numbers of gene copy equivalents using the housekeeping gene *glyA* as an internal standard. Approximate equal addition of bacterial cells in the ELISA set-up is assessed, as described in *Materials and Methods*.

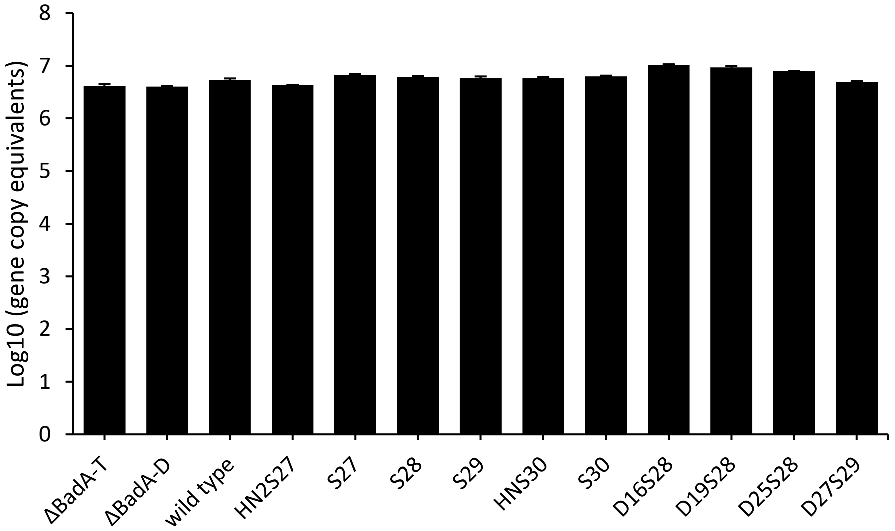

Figure S3. Analysis of the specificity of anti-BadA-DALL IgG antibodies. (A) Western Blotting: anti-BadA-DALL IgG antibodies react specifically with *B. henselae* strains containing BadA constructs with the targeted DALL motif sequence (RHEKSKLEKGASKAI). Negative control strains ΔBadA-T, S28, S29, and S30 (missing the targeted DALL motif sequence) are not detected. The order of the lanes of the original nitrocellulose membrane has been rearranged *in silico* (dotted lines). **(B)** ELISA: anti-BadA-DALL IgG antibodies react specifically with Fn-bound *B. henselae* strains that contain BadA constructs with the targeted DALL motif sequence (wild type, S27, and D27S29) but not with strains that miss the targeted DALL motif sequence (ΔBadA-T, D16S28, and D25S28). Strain D19S28 contains a highly similar targeted DALL motif sequence only differing two amino acids and likewise reacts with the anti-BadA-DALL IgG antibodies. Negative control 1: no bacteria, negative control 2: wild type *B. henselae* without the addition of Fn. **(C)** Fluorescence microscopy: anti-BadA-DALL IgG antibodies react specifically with *B. henselae* strains containing BadA constructs with the targeted DALL motif sequence, indicated by a green halo around the DAPI-stained intracellular DNA. Negative control strains ΔBadA-T, S28, S29, and S30 (missing the targeted DALL motif sequence) are not stained by the antibodies. Scale bar: 5 µm.

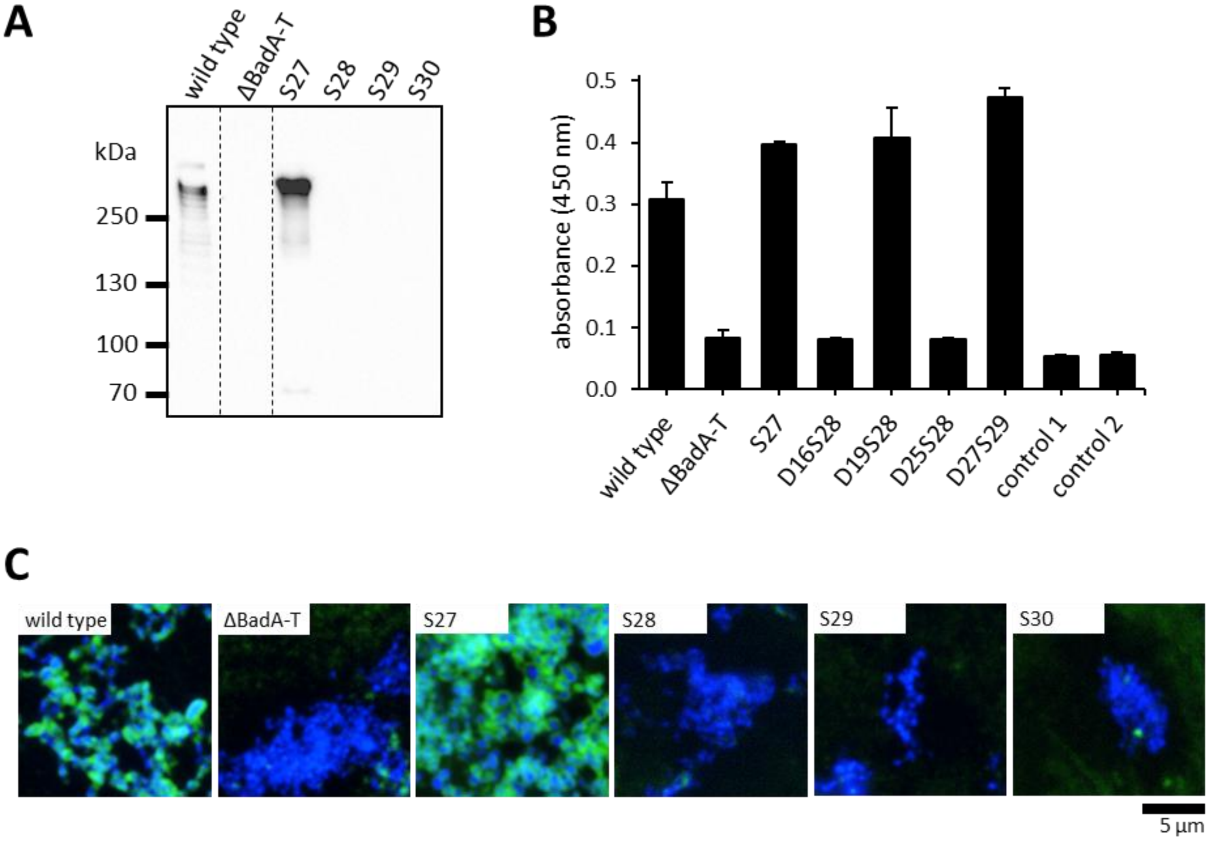

Table S1: Primers used in this study. Underlined regions create overhangs in resulting PCR-fragments.

primer	5'-3' sequence
S28domains_Fw	CTCTAGAACTAGTGGATCCCGAATTCCTGAATTTAGAGAGTG
S28domains_Rv	GTATCGATAAGCTTGATATCGAATTCTTTTTCGTAGAAACAAGAG
pBBR1MCS-5_Fw	GTTTCTACGAAAAAGAATTCGATATCAAGCTTATCGATACC
pBBR1MCS-5_Rv	CTCTCTAAATTCAGGAATTCGGGATCCACTAGTTCTAGAGC
pBBR1MCS-5_GA_Fw	CTCACTATAGGGCGAATTGG
pBBR1MCS-5_GA_Rv	GAGTTAGCTCACTCATTAGGC
BadA1_Fw	CGTTCCCAATTTGACCAC
BadA2_Fw	TATTCAATGAGCAATATGCTTGCGACC
BadA3_Fw	AGTTGGTACGTCTTTCACC
glyA_Fw	GACAGGAAAATGTGCCGAAT
glyA_Rv	GCAGGTGAACCAAGACGAAT

Table S2. Human patient sera used in this study.

patient serum	IgG titre *B. henselae*	patient diagnosis
1	20,180	lymphadenopathy (cat scratch disease)
2	10,240	unknown
3	10,240	unknown
4	5,120	endocarditis
5	5,120	unknown
6	5,120	splenomegaly, hepatomegaly, and lymphadenopathy
7	5,120	unknown
8	5,120	lymphadenopathy (cat scratch disease)
9	2,560	lymphadenopathy (cat scratch disease)
10	1,280	unknown
11	640	lymphadenopathy (cat scratch disease)
12	640	endocarditis (suspected)
13	-	unknown - negative control
14	-	unknown - negative control

Table S3. Comparative overview of cross-linking mass spectrometry identified interaction sites between BadA and Fn (9). *B. henselae* mutant strains are displayed according to their ELISA-based Fn binding capacity (high to low; **Figure 5A**) that is given underneath each strain. The location (motif) within BadA of all identified BadA-Fn interaction site is given underneath in italics and is visualised in **Figure 2A** (highlighted in light blue). Identified sequences are listed in the main table if they appear in the corresponding truncated BadA mutant. Differences in amino acid residues between the identified BadA-Fn interaction site and the present sequence are highlighted in grey.

**strain**<br>*ELISA read-out*	**identified cross-linking mass spectrometry BadA-Fn interaction sites**					
				**LEKGASKATQENSKITYLLDGDVSK**		
	**TVNGEGKEEEK**<br>*FGG motif*	**VNNNVTNKFNELTQSITNVTQQVK**<br>*coiled-coil*	**VEDKLTEAVGK**<br>*coiled-coil*	**LEKGASK**<br>*DALL motif*	**GASKATQENSK**<br>*DALL motif*	**ATQENSKITYLLDGDVSK**<br>*DALL-neck motif*
**S27**<br>*0.745*	TVNGEGKEEEQ	VNNNVTNKLSELTQNITTVTQQVK		LEKGASK	GASKAIQENSK	AIQENSKITYLLDGDVSK
**D19S28**<br>*0.713*	TVNGEGKEEEQ	VNNNVTNKFNELTQSITNVTQQVK		LEKGVSK	GVSKATQENSK	ATQENSKITYLLDGDISK
**D16S28**<br>*0.545*	TVNGEGKEEEQ<br>TVNGEGKEEEQ					
**D27S29**<br>*0.544*		VNNNVTNKLSELTQNITTVTQQVK		LEKGASK	GASKAIQENSK	AIQENSKITYLLDGDVSK
**D25S28**<br>*0.383*	TVNGEGKEEEQ		VEDKLTEAVGK			
**S28**<br>*0.334*	TVNGEGKEEEQ					
